# Supplementary figures and images for: Continuous positive airway pressure to reduce the risk of early peripheral oxygen desaturation after onset of apnoea in children: A double-blind randomised controlled trial
Source: PLoS One. 2021 Oct 1;16(10):e0256950. doi: 10.1371/journal.pone.0256950 (PMC8486132; doi:10.1371/journal.pone.0256950)

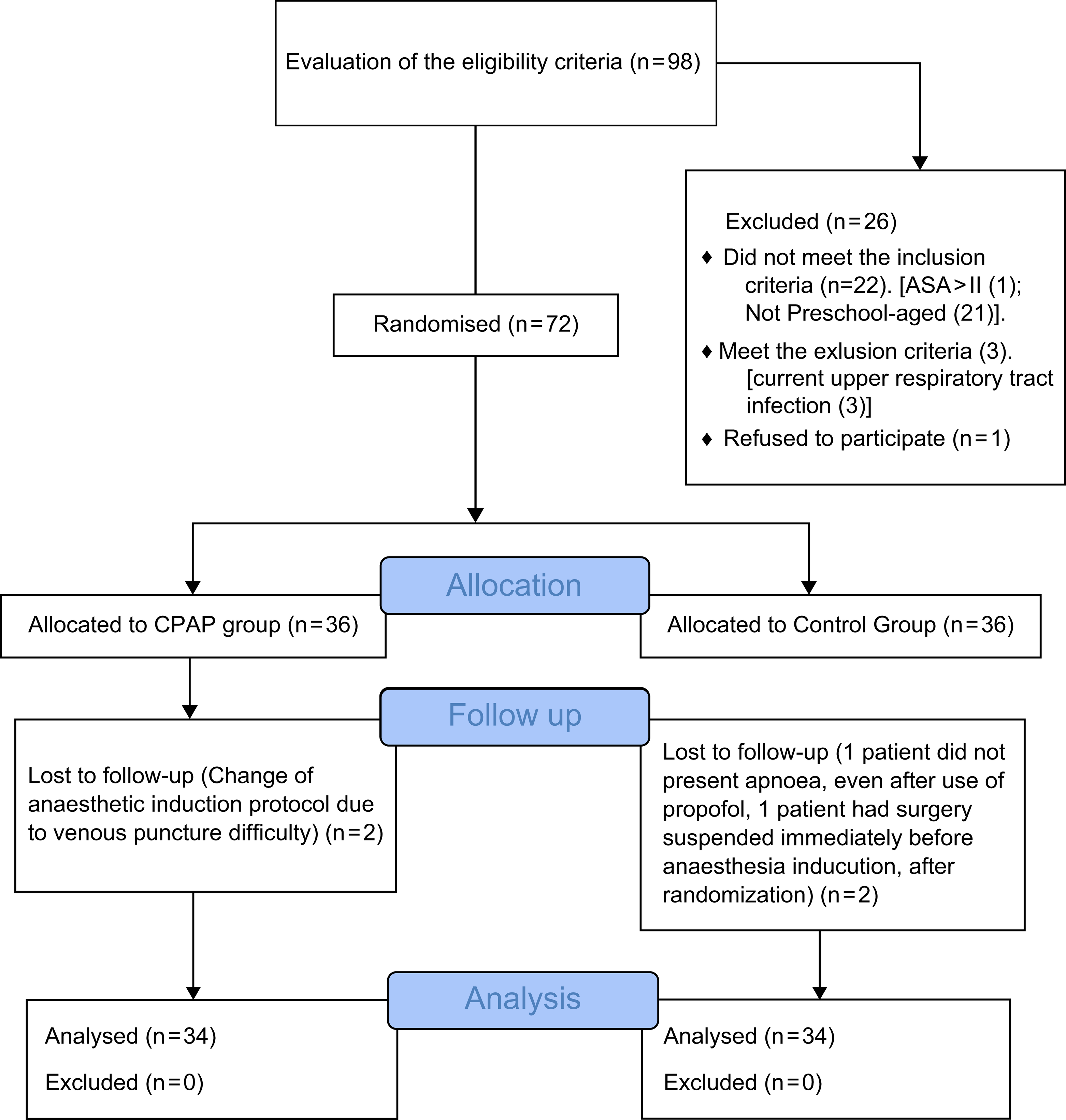

Supplement: S1 Fig — (TIF) [file pone.0256950.s001.tif]

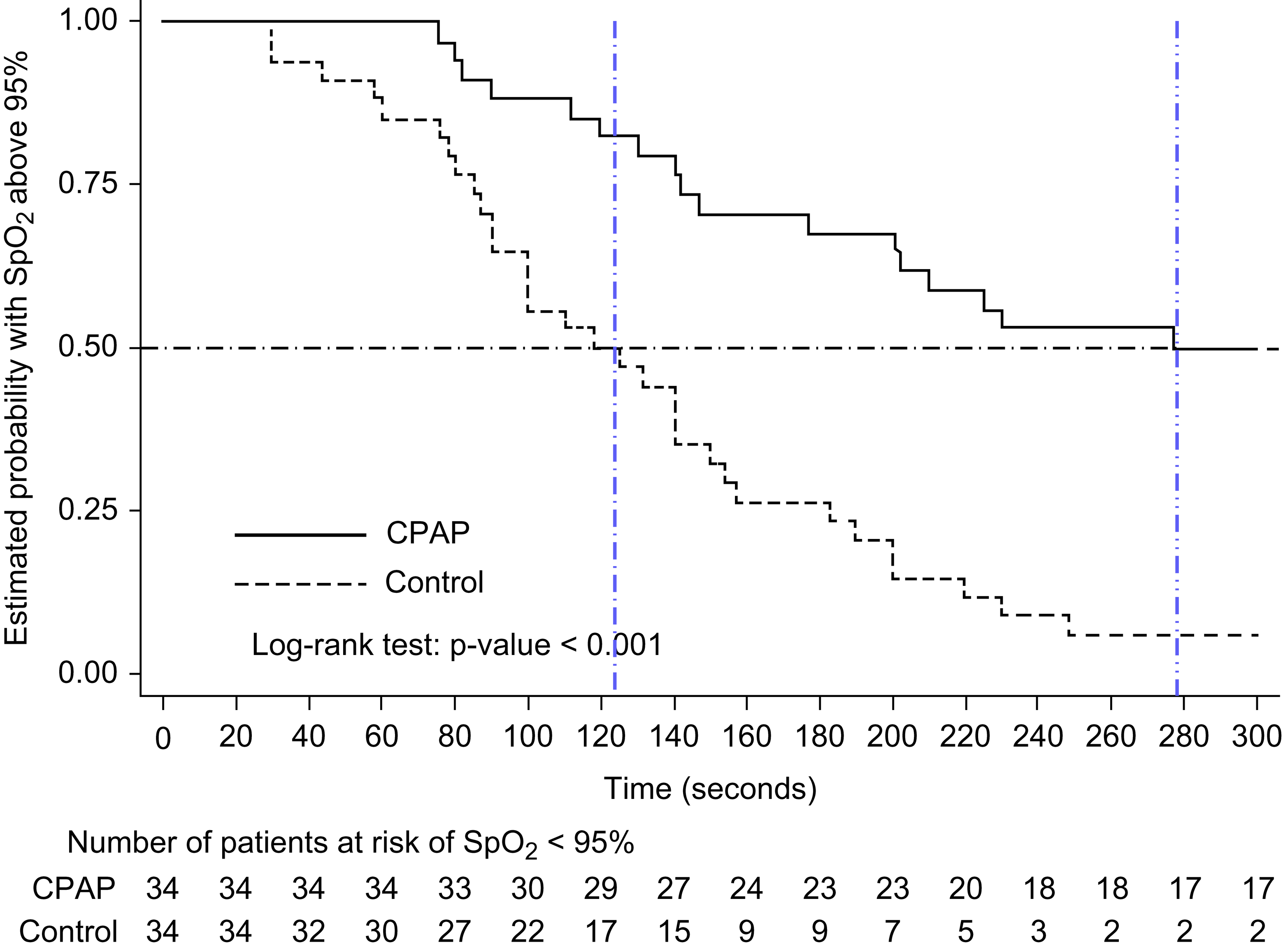

Supplement: S2 Fig — Occurrence of an SpO2 of 95% during a 5-min follow-up in children with CPAP ventilation and passive CPAP oxygenation (10 cmH2O) or no positive airway pressure (0 cmH2O) during anaesthesia induction for elective surgery showing a significant difference between the survival curves (log-rank test; p<0.001). (TIF) [file pone.0256950.s002.tif]

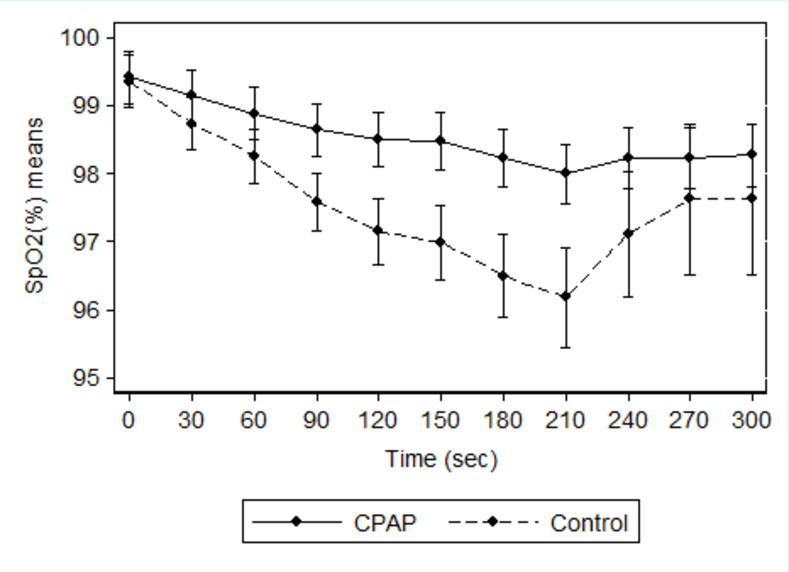

Supplement: S3 Fig — Mean SpO2 values in children with either CPAP ventilation and passive CPAP oxygenation (10 cmH2O) or no positive airway pressure (0 cmH2O) during anaesthesia induction for elective surgery and their respective confidence intervals in the two-group interaction (p ranging from 0.047 to <0.001 for the interaction between the curves in the 60–210 s interval). (TIF) [file pone.0256950.s003.tif]
