# Supplementary material for: Continuous positive airway pressure to reduce the risk of early peripheral oxygen desaturation after onset of apnoea in children: A double-blind randomised controlled trial
Source: PLoS One. 2021 Oct 1;16(10):e0256950. doi: 10.1371/journal.pone.0256950 (PMC8486132; doi:10.1371/journal.pone.0256950)
Supplement: S2 File — (PDF) [file pone.0256950.s005.pdf]

Instituto de Medicina Integral Prof. Fernando Figueira

PRESSÃO POSITIVA CONTÍNUA NAS VIAS AÉREAS DURANTE A INDUÇÃO DE ANESTESIA GERAL PARA CIRURGIA PEDIÁTRICA ELETIVA: ENSAIO CLÍNICO RANDOMIZADO

Projeto apresentado como parte dos requisitos para obtenção do grau de Mestre em Saúde Integral

Mestrando: Jayme Marques dos Santos Neto

Orientadora: Flávia Orange

Co-orientadoras: Lívia Andrade e Rebeca Gonelli

Linha de Pesquisa: Estudos epidemiológicos, clínicos e cirúrgicos dos agravos prevalentes na infância e adolescência

Fevereiro/2019

Mestrando: Jayme Marques dos Santos Neto

Médico Anestesiologista do Hospital das Clínicas de Pernambuco

RG: 6353506 – SDS-PE   CPF: 043092024-54

Tel: 996212977

E-mail: jaymemed@yahoo.com.br

Orientadora:

Profª Drª Flávia Augusta de Orange Lins da Fonseca e Silva

Anestesiologista do Instituto de Medicina Integral Prof. Fernando Figueira – IMIP; Profª Drª da Faculdade Pernambucana de Saúde - FPS.

Telefone: 81 994197979

E-mail: orangeflavia@gmail.com

Coorientadoras:

Profª Drª. Lívia B. Andrade

Doutora em Saúde Materno Infantil pelo IMIP

Coordenadora de Tutor do curso de Fisioterapia da Faculdade Pernambucana de Saúde - FPS.

Coordenação da Residência em Fisioterapia Respiratória do IMIP

Telefone: 81 991548350

Email: liviaposimip@yahoo.com.br

Drª. Rebeca Gonelli

RESUMO

CENÁRIO: Hipoxemia é um dos eventos adversos mais comuns durante a indução de anestesia geral e pode culminar com complicações mais graves como parada cardíaca e morte. Os pacientes pediátricos, por características anatômicas e fisiológicas, são mais propensos a desenvolver queda dos níveis de saturação da hemoglobina. Algumas estratégias preventivas são utilizadas nessa fase da anestesia para reduzir as chances de ocorrência desse evento adverso. A pressão positiva contínua nas vias aéreas (CPAP) pode ser útil durante a indução anestésica em retardar a queda dos níveis de oxigênio no sangue por aumentar as reservas corporais desse gás. OBJETIVOS: Avaliar a efetividade do CPAP durante a indução anestésica em aumentar o tempo de apneia até que a saturação da hemoglobina caia a 95% em crianças submetidas a anestesia geral para cirurgia eletiva. MÉTODOS: Ensaio clínico, fase III, paralelo, randomizado a ser desenvolvido no hospital das Clínicas de Pernambuco. Os pacientes (72) serão divididos em dois grupos (36 em cada) nos quais todos ventilarão espontaneamente: o grupo C receberá CPAP e o grupo A utilizará o sistema aberto. Serão incluídas crianças em idade pré-escolar com estado físico, segundo a sociedade americana de anestesia, I ou II e que sejam candidatas a cirurgia eletiva sob anestesia geral. Os critérios de exclusão serão doença pulmonar parenquimatosa pré-existente, crianças cianóticas ou com saturação da oxihemoglobina menor que 95% antes da indução anestésica e história recente (<4 semanas) ou vigência de infecção do trato respiratório superior. A análise estatística descritiva será realizada através de medidas de tendência central e de dispersão para as variáveis quantitativas e através distribuição de frequências para as variáveis qualitativas. ASPECTOS ÉTICOS: Este trabalho respeitará os direitos humanos, os

princípios da bioética, a resolução 466/2012 do Conselho Nacional de Saúde e a declaração de Helsinque. A coleta de dados só será iniciada após submissão e aprovação pelo comitê de ética em pesquisa. PALAVRAS-CHAVE: Pressão Positiva Contínua nas Vias Aéreas, Hipóxia, Anestesia Geral

ABSTRACT

SCENARIO: Hypoxemia is one of the most common adverse events during the induction of general anesthesia and may culminate with more serious complications such as cardiac arrest and death. Pediatric patients, due to their anatomical and physiological characteristics, are more likely to develop hemoglobin desaturation levels. Some preventive strategies are used during this period to reduce the chances of occurrence of adverse event. Continuous Positive Airway Pressure (CPAP) may be useful during anesthetic induction in delaying the drop in oxygen levels in the blood by increasing this body gas reserves. OBJECTIVES: To assess the effectiveness of CPAP during anesthetic induction in increasing apnea time until hemoglobin saturation falls to 95% in children undergoing general anesthesia for elective surgery. METHODS: Phase III, parallel, randomized clinical trial to be developed at the Hospital das Clínicas de Pernambuco. Patients (72) are divided into two groups (36 in each) in which all patients will spontaneously ventilate: group C will receive CPAP and group A will use the open system. Children of pre-school age with physical status I or II, according to the American Anesthesia Society, candidates for elective surgery under general anesthesia will be included. Patients with pre-existing parenchymal lung disease, cyanotic children or patients with oxyhemoglobin saturation <95% prior to anesthetic induction and recent history (<4 weeks) or active upper respiratory tract infection will be excluded. The descriptive statistical analysis will be carried out through measures of central tendency and dispersion for quantitative variables and via distribution of frequencies for qualitative variables. ETHICAL ASPECTS: This work will respect human rights, principles of bioethics, the resolution 466/2012 of the National Health Council and the

statement from Helsinki. Submission and approval by the research ethics committee is required prior to data collection. KEY WORDS: Continuous Positive Airway Pressure, Hypoxia, General Anesthesia

SUMÁRIO

I. INTRODUÇÃO 1

II. JUSTIFICATIVA 8

III. HIPÓTESES 10

IV. OBJETIVOS 11

V. MÉTODOS 12

5.1. Desenho do estudo 12

5.2. Local do estudo 12

5.3. Período do estudo 12

5.4. População do estudo 12

5.5. Amostra 12

5.6. Critérios e procedimentos para seleção, captação e acompanhamento dos participantes 13

5.7. Fluxograma de captação e acompanhamento dos participantes 16

5.8. Termos, Variáveis e Conceitos 17

5.9. Procedimentos, testes, técnicas e exames 23

5.10. Procedimentos para coleta dos dados 25

5.11. Processamento e análise dos dados 26

5.12. Aspectos éticos 27

5.13. Conflitos de interesse 28

VI. PLANO DE RESULTADOS 29

VII. ORÇAMENTO 33

VIII. CRONOGRAMA...34

IX. REFERÊNCIAS 35

APÊNDICE 1 - LISTA DE CHECAGEM 40

APÊNDICE 2 - TERMO DE CONSENTIMENTO LIVRE E ESCLARECIDO 41

APÊNDICE 3 – FORMULÁRIO 46

ÍNDICE DE FIGURAS

Figura 1 Fluxograma de captação e acompanhamento dos participantes

LISTA DE SIGLAS, SÍMBOLOS E ABREVIATURAS

ASA	Sociedade Americana de Anestesiologia
CEP	Comitê de Ética em Pesquisa
cmH_2O	Centímetro de Água
CO_2	Dióxido de carbono
CPAP	Pressão Positiva Contínua na Via Aérea
CRF	Capacidade Residual Funcional
FiO_2	Fração inspirada de Oxigênio
G	Gauge
HC-UFPE	Hospital das Clínicas da Universidade Federal de Pernambuco
g/m^2	Grama por metro quadrado
IMIP	Instituto de Medicina Integral Prof. Fernando Figueira
l/min	Litros por minuto
mg/kg	Miligramas por quilograma
NNH	Número Necessário para Tratar ou se obter um malefício
NNT	Número Necessário para Tratar e obter um benefício
O_2	Oxigênio
PaO_2	Pressão arterial de Oxigênio
$PaCO_2$	Pressão arterial de dióxido de carbono
PCR	Parada Cardiorrespiratória
PEEP	Pressão Expiratória Final na Via Aérea
TCLE	Termo de Consentimento Livre e Esclarecido
VO_2	Consumo de oxigênio
V/Q	Relação ventilação/perfusão

I. INTRODUÇÃO

Anestesia geral pode ser definida como um estágio transitório de inconsciência induzido por drogas através de suas ações em receptores moleculares[1,2]. Pouco ainda é conhecido sobre seus mecanismos, mas supõe-se que esse estado seja um fenômeno adaptativo assim como o sono[1]. Trata-se de uma condição reversível que apresenta padrões comportamentais e fisiológicos específicos (inconsciência, amnésia, analgesia e acinesia). Consiste de três fases: indução, manutenção e emergência ou despertar[2].

A indução anestésica é o período em que há o início da administração das drogas hipnóticas (venosas, inalatórias ou uma combinação de ambas)[2,3]. Nessa fase, um padrão respiratório irregular progride para apneia. Assistência ventilatória é então iniciada pelo anestesiologista, normalmente através de máscara facial e bolsa reservatório[2,4].

Indução inalatória é uma técnica segura, factível e bem aceita pelos pacientes, utilizada amplamente em crianças ou, excepcionalmente, em adultos com acesso venoso difícil. Entretanto, não é um método isento de riscos. Complicações como tosse, laringoespasmo, salivação, falha de indução e apneia voluntária podem aumentar a morbidade do procedimento anestésico[3,5].

Os efeitos da anestesia geral sobre o sistema respiratório são bem estabelecidos, dentre eles, destaca-se a maior predisposição para obstrução e colapso da via aérea. Seus mecanismos não são claramente conhecidos apesar de algumas associações serem documentadas (relaxamento da musculatura ventilatória, relação dose-efeito do nível de anestesia e comprometimento da patência da via aérea)[6,7]. A perda do tônus da

musculatura respiratória, decorrente da indução anestésica, está relacionada a colapso das vias aéreas inferiores, reduzindo a capacidade residual funcional (CRF)[7].

Outra explicação aditiva ao colapso das vias aéreas é o impacto farmacológico das drogas utilizadas para a sedação e hipnose nos processos que controlam tanto as vias eferentes motoras da musculatura faríngea como a aferência dos mecanorreceptores[6].

Os anestésicos inalatórios exacerbam o colapso dinâmico das vias aéreas, em especial no palato mole, tendo associação direta com a dose administrada da droga. O sevoflurano atua reduzindo a atividade fásica inspiratória do músculo genioglosso sem comprometer sua atividade tônica. Mesmo em níveis sedativos, o sevoflurano modifica as dimensões das vias aéreas, reduzindo sua patência especialmente na inspiração[6].

Assim como os fármacos inalatórios, os anestésicos venosos também apresentam ação sobre o sistema respiratório. O propofol reduz a área de secção transversal da via aérea superior, redução que se mostrou máxima na base da língua quando doses sedativas são utilizadas. A indução com esse agente reduz a atividade eletromiográfica do músculo genioglosso[6].

Durante o período de apnéia que se segue à indução da anestesia geral, as reservas de oxigênio (O_2) vão sendo consumidas o que pode resultar em hipoxemia[8]. A dessaturação da oxihemoglobina é mais rápida em pacientes com capacidade reduzida de transporte de O_2, ou seja, naqueles com diminuição da CRF, pressão parcial de oxigênio (PaO_2), conteúdo arterial de oxigênio e débito cardíaco, ou aumento do consumo de oxigênio (VO_2)[9].

Anestesia geral aparece como fator de risco maior de mortalidade em pacientes pediátricos cirúrgicos, bem como problemas no manejo da via aérea em pacientes com

comorbidades parece adicionar risco de vida à essa população[10]. Os pacientes pediátricos, por sua vez, apresentam com mais frequência episódios de dessaturação durante a indução (4-10%)[5,8,11,12]. Crianças submetidas a uma maior quantidade de tentativas de intubação traqueal, consideradas portadoras de via aérea difícil, estão sob maior risco de dessaturação que, se não tratada, evolui naturalmente para hipoxemia[12]. A ocorrência dessa durante a manipulação das vias aéreas em crianças pode vir acompanhada de complicações ainda mais graves como parada cardiorrespiratória (PCR) e morte[11].

A queda da saturação da oxihemoglobina a níveis menores ou iguais a 95% induz a alterações em parâmetros hemodinâmicos como o índice sistólico [razão entre o índice cardíaco (volume sistólico x frequência cardaca/superfície corporal) e frequência cardíaca] o que sinaliza uma redução da função do coração[13].

Dados de estudo realizado em hospital terciário num período de cinco anos (12.158 cirurgias) documentam os eventos respiratórios como responsáveis por 29% das paradas cardíacas perioperatórias em crianças. Essa participação sobe para mais da metade (56%) quando são analisados os dados relacionados de alguma maneira à anestesia. Todas as PCR atribuídas exclusivamente ao ato anestésico se deram na indução[14].

Outro estudo (10.649 anestesias) realizado através de questionário com dados de um período de seis anos aponta os problemas no manejo de via aérea como a maior causa de PCR relacionada à anestesia[15].

Características anatômicas próprias podem contribuir para ocorrência de hipoxemia em crianças no perioperatório, tais como: 1) cabeça e língua

proporcionalmente grandes; 2) hipertrofia de adenóides e amígdalas; 3) hipofaringe menor e mais estreita; 4) laringe mais alta na altura do pescoço; 5) cordas vocais inclinadas, e não em ângulo reto; 6) epiglote em formato de "U" invertido; e 7) menor raio das vias aéreas em comparação com os adultos, o que impõe mais resistência ao fluxo de ar de acordo com a lei de Poiseulle ($R = 8\eta L/\pi r^4$). Todos esses fatores implicam em desafios diários à manipulação da via aérea, seja na ventilação ou durante a intubação[16]. Em lactentes, ocorre fechamento das vias aéreas durante a indução da anestesia geral, primariamente na direção antero-posterior, sendo uniforme em toda a faringe, o que muda em crianças mais velhas nas quais a epiglote é o ponto de maior estreitamento[6].

As características fisiológicas dos pacientes pediátricos, tais como, menor CRF, maior VO_2, maior produção de gás carbônico (CO_2) e incidência maior de complicações respiratórias durante o período de indução quando há interrupção da oferta de O_2, também contribuem para a queda na saturação de oxigênio pela hemoglobina[9,11,16,17]. A idade tem ainda correlação linear com a duração da apneia antes da dessaturação da oxihemoglobina, assim como quanto menor o peso do paciente maior a incidência de episódios graves do evento[8,11]. Os riscos de obstrução e dessaturação parecem ser maiores em crianças até três anos[6].

Uma das estratégias de que o médico anestesiologista lança mão para a prevenção dessa complicação é a pré-oxigenação, oferta de O_2 em níveis acima dos habitualmente respirados pelo paciente com o objetivo de aumentar seus estoques. Essa estratégia permite prolongar o período de tempo antes da dessaturação da oxihemoglobina[9,11,18]. Em modelos teóricos, as reservas fisiológicas corporais de O_2

(pulmão, plasma e hemoglobina) podem aumentar em mais de duas vezes e meia quando a fração inspirada daquele gás (FiO_2) é igual a 1. Esse aumento se dá principalmente às custas da fração alveolar na CRF, principal reservatório de oxigênio do corpo[8,17]. Essa, associada a VO_2 e débito cardíaco, é responsável pela disponibilidade de O_2 ao paciente[8].

Pré-oxigenação, a despeito dos benefícios, também pode contribuir para a ocorrência de dessaturação da oxihemoglobina. Microatelectasias e distúrbio da relação ventilação/perfusão (V/Q) são documentados durante indução de anestesia sob diferentes FiO_2. Manobras de recrutamento alveolar e utilização de pressão expiratória final na via aérea (PEEP) podem reverter e prevenir, respectivamente, a ocorrência daqueles eventos[7,8]. Outras estratégias preventivas, como oxigenação apnéica, também vem sendo estudadas, mas não se sabe ainda qual técnica é a ideal[19,20].

Uma variação de ventilação não invasiva, a Pressão Positiva Contínua na Via Aérea (CPAP) é um modo ventilatório no qual o paciente respira espontaneamente através de um circuito pressurizado[21]. Em pacientes portadores de apneia obstrutiva do sono, na qual seu uso já é bem estabelecido, foram evidenciados benefícios como melhora da sonolência e gravidade da doença, melhora dos desfechos cardiovasculares, além de redução na pressão arterial, e efeitos adicionais indiretos como melhora na resistência insulínica em não diabéticos[22-24].

Do ponto de vista ventilatório, seus benefícios são demonstrados pela melhora na troca gasosa alveolar, minimização da formação de atelectasia e aumento tanto da capacidade residual funcional quanto do volume corrente[21]. Melhoras na saturação

periférica de oxigênio, pico de fluxo respiratório e redução tanto na frequência quanto no trabalho respiratório já foram evidenciados em pacientes durante crise asmática[25].

Na população pediátrica, a aplicação do CPAP é amplamente estudada em pacientes com bronquiolite como alternativa à ventilação mecânica controlada em decorrência de seus efeitos nas pequenas vias aéreas (abertura alveolar, prevenção de atelectasia e aumento da capacidade residual funcional)[26,27]. Estudo realizado com pacientes entre três meses a cinco anos de vida com desconforto respiratório mostrou também um potencial benefício através da redução da frequência respiratória[28].

Existe evidência de que a CPAP pode ser eficiente em minimizar os efeitos deletérios da pré-oxigenação sob altas FiO_2 através da manutenção do volume pulmonar[7]. Seu uso na ventilação durante a indução de anestesia geral ainda necessita de estudos bem conduzidos para sustentar sua prática como rotina, mas pesquisas em adultos mostram resultados encorajadores[29,30].

Em pacientes sem comorbidades, candidatos a cirurgia de grande porte, foram significativos o maior tempo de apneia antes da dessaturação e maiores valores de PaO_2 naqueles que receberam CPAP durante a indução anestésica. O uso de CPAP também reduziu o tempo de retorno ao valores basais normais de saturação após apneia[29].

O uso de CPAP também esteve relacionado a valores maiores de PaO_2 e menores de pressão arterial de dióxido de carbono ($PaCO_2$) em obesos na indução de anestesia. Não ocorreram episódios de hipoxemia nesses pacientes, diferentemente daqueles que não utilizaram a técnica de pressão positiva contínua[30].

O objetivo desse trabalho é avaliar a efetividade do CPAP durante a indução anestésica em aumentar o tempo de apneia até que a saturação da hemoglobina caia a 95% em crianças submetidas a anestesia geral para cirurgia eletiva.

II. JUSTIFICATIVA

A indução anestésica é um período critico que requer atenção especial e isso se deve principalmente ao período de apneia que segue o uso das drogas hipnóticas. Sabe-se que curtos períodos de apneia não acarretam grandes repercussões para o paciente, mas em situações de ventilação-intubação difícil a ocorrência de apneia por um período prolongado pode levar a ocorrência de dessaturação da oxihemoglobina.

Dessa forma, avaliar a efetividade de manobras que possam melhorar a reserva ventilatória é extremamente relevante. Pode significar melhoria na assistência e pode trazer evidências científicas importantes para a comunidade científica

Após pesquisa nas bases de dados LILACS, Scielo, Bireme, PuBmed e Biblioteca Cochrane, poucos estudos foram encontrados na população adulta. Não encontramos estudo conduzido na população pediátrica. Trata-se, portanto, de um estudo novo que pode trazer avanços na assistência a pacientes pediátricos submetidos a anestesia, aumentando a segurança da prática anestésica.

É factível, visto que de acordo com o cálculo amostral (72 pacientes) e levando em consideração o número de cirurgias realizadas por mês na clinica cirúrgica pediátrica, não será difícil alcançar o número de sujeitos necessário para responder a pergunta de pesquisa. Além disso, os pesquisadores envolvidos apresentam *expertise* em anestesia pediátrica e na condução ventilatória.

Vale ressaltar, que este estudo respeitará os direitos humanos, os princípios da bioética, a resolução 466/2012 do Conselho Nacional de Saúde e a declaração de

Helsinque, e pela inovação do tema será possivelmente publicado em revista de elevado impacto na área.

III. HIPÓTESES

3.1. Hipótese primária

- O uso de CPAP na ventilação pulmonar de pré-escolares durante a indução de anestesia geral para cirurgia eletiva retarda a ocorrência de dessaturação da oxihemoglobina durante período de apneia.

3.2. Hipóteses secundárias

- Valores de saturação de oxihemoglobina na oximetria de pulso em pacientes apnéicos em períodos semelhantes durante indução anestésica são maiores naqueles que utilizarem CPAP;

- Tempo para recuperação dos níveis normais de saturação de oxihemoglobina na oximetria de pulso após período de apneia é menor em pacientes que utilizarem CPAP;

- A frequência de complicações (laringoespasmo, hipoxemia, bradicardia, parada cardiorrespiratória, morte) é menor em pacientes que utilizarem CPAP.

IV. OBJETIVOS

4.1. Objetivo geral

Avaliar a efetividade do CPAP durante a indução anestésica em aumentar o tempo de apneia até que a saturação da hemoglobina caia a 95% em crianças submetidas a anestesia geral para cirurgia eletiva.

4.2. Objetivos específicos

Em crianças submetidas a anestesia geral para cirurgia eletiva que serão submetidas durante a indução anestésica a ventilação com CPAP ou com circuito circular padrão, comparar:

DESFECHO PRIMÁRIO:

- O tempo entre o início da apneia e a queda da saturação da oxihemoglobina a 95% entre os grupos.

DESFECHO SECUNDÁRIO:

1. Os valores de saturação de oxihemoglobina na oximetria de pulso durante a indução anestésica em diferentes momentos entre os grupos;
2. O tempo para recuperação dos níveis da saturação da oxihemoglobina na oximetria de pulso pré-apneia entre os grupos;
3. A frequência de complicações (laringoespasmo, hipoxemia, bradicardia, parada cardiorrespiratória, morte) entre os grupos.

V. MÉTODOS

5.1. Desenho do estudo

Trata-se de um ensaio clínico randomizado, fase III, paralelo, em pacientes pediátricos submetidos a cirurgias eletivas.

5.2. Local do estudo

O estudo será desenvolvido no bloco cirúrgico do Hospital das Clínicas da Universidade Federal de Pernambuco (HC-UFPE).

O serviço de cirurgia pediátrica do HC-UFPE realiza cerca de 15-20 cirurgias por semana em caráter eletivo; a equipe é composta por seis cirurgiões e três médicos residentes; e dispõe de uma sala no bloco cirúrgico em quatro turnos semanais nos quais realiza procedimentos em crianças desde o nascimento até os 18 anos.

5.3. Período do estudo

O estudo será realizado no período de janeiro de 2018 a junho de 2018.

5.4. População do estudo

Pacientes pediátricos pré-escolares submetidos a cirurgia eletiva no bloco cirúrgico do Hospital das Clínicas.

5.5. Amostra

5.5.1. Amostragem

Será obtida uma amostra não probabilística de conveniência, composta pelas crianças em fase pré-escolar que serão submetidas a anestesia geral para cirurgias eletivas, obedecendo aos critérios de inclusão e exclusão do estudo.

5.5.2. Tamanho da Amostra

O cálculo do tamanho da amostra foi realizado no programa Openepi, versão 3.01 (Dean AG, Sullivan KM, Soe MM. OpenEpi: Open Source Epidemiologic Statistics for Public Health, Versão. www.OpenEpi.com, atualizado 2013/04/06, acessado 2017/07/11), usando diferença de médias. O primeiro parâmetro utilizado foi a média de tempo em apneia que os pacientes expostos à intervenção levaram para atingir uma saturação de oxigênio na oximetria de pulso de 95% (166 ± 47 segundos)[20]. O segundo parâmetro foi a média de tempo em apneia que os pacientes não expostos à intervenção levaram para atingir uma saturação de oxigênio na oximetria de pulso de 95% (131 ± 39 segundos)[20]. Considerando um nível de significância de 5% e um poder de 90%, serão necessários 64 pacientes (32 em cada grupo). Entretanto, prevendo-se eventuais perdas por exclusão pós-randomização (em torno de 10%), esse número será aumentado para 72 (36 em cada grupo).

5.5.3. Procedimento para randomização

A tabela de randomização será gerada no computador, utilizando-se o programa Random Software Allocation. Após a randomização, serão então preparados envelopes opacos numerados sequencialmente de 1 a 72 de acordo com a tabela de números randômicos. Será respeitada a ocultação da alocação.

5.6. Critérios e procedimentos para seleção, captação e acompanhamento dos participantes

5.6.1. Critérios de inclusão

- Crianças em idade pré-escolar;
- ASA I ou II;
- Crianças submetidas a anestesia geral para cirurgia eletiva;

5.6.2. Critérios de exclusão

- Doença pulmonar parenquimatosa pré-existente;
- Crianças cianóticas ou com saturação da oxihemoglobina menor que 95% antes da indução anestésica;
- História recente (<4 semanas) ou vigência de infecção do trato respiratório superior;

5.6.3. Procedimentos para Captação e Acompanhamento das Participantes

A captação dos participantes será realizada por um aluno de iniciação científica que não participará da coleta de dados. Ele ficará unicamente responsável por captar os participantes, aplicar os critérios de elegibilidade utilizando uma lista de checagem (Apêndice 1) e solicitar a assinatura do TCLE. Essa etapa acontecerá na entrada do bloco cirúrgico onde os pacientes e acompanhantes, vindos do ambulatório após pesagem e medição, aguardam a cirurgia. Posteriormente, entregará ao pesquisador principal o envelope referente ao participante onde constará no interior o grupo a que ele foi alocado.

A alocação será feita por envelopes sequencialmente numerados, de outra forma idênticos, selados, cada um contendo um papel de 2 polegadas por 2 polegadas com um código escrito que designará o grupo intervenção ou o grupo comparativo. Não haverá diferenças detectáveis em tamanho ou peso entre os envelopes do grupo intervenção e os envelopes do grupo comparativo. Os envelopes serão opacos e abertos sequencialmente somente depois de escritas neles as informações referentes ao pacientes aos quais foram designados. A abertura do envelope ocorrerá antes da entrada do paciente na sala de cirurgia para que o cenário do estudo seja montado.

O pesquisador principal será responsável por todo o procedimento, juntamente com o anestesista responsável pela cirurgia. A coleta de dados por sua vez será realizada por um aluno de iniciação científica responsável apenas pela coleta dos dados sem conhecimento do grupo a que o paciente será alocado (sistema de CPAP será selecionado antes da entrada do aluno na sala para coleta).

Será preenchido um fluxograma (CONSORT) com o progresso do estudo ao longo das fases de um estudo de intervenção em paralelo de dois grupos (seleção dos participantes, alocação de intervenção, acompanhamento e análise de dados) (Figura 1).

Todos os prontuários dos participantes envolvidos no estudo serão identificados por meio de etiquetas autocolantes contendo o nome da pesquisa, número do registro, número de identificação da paciente no estudo e o grupo em que foi alocado.

5.7. Fluxograma de captação e acompanhamento dos participantes

O fluxograma de captação e acompanhamento dos participantes da pesquisa está demonstrado na figura 1, abaixo.

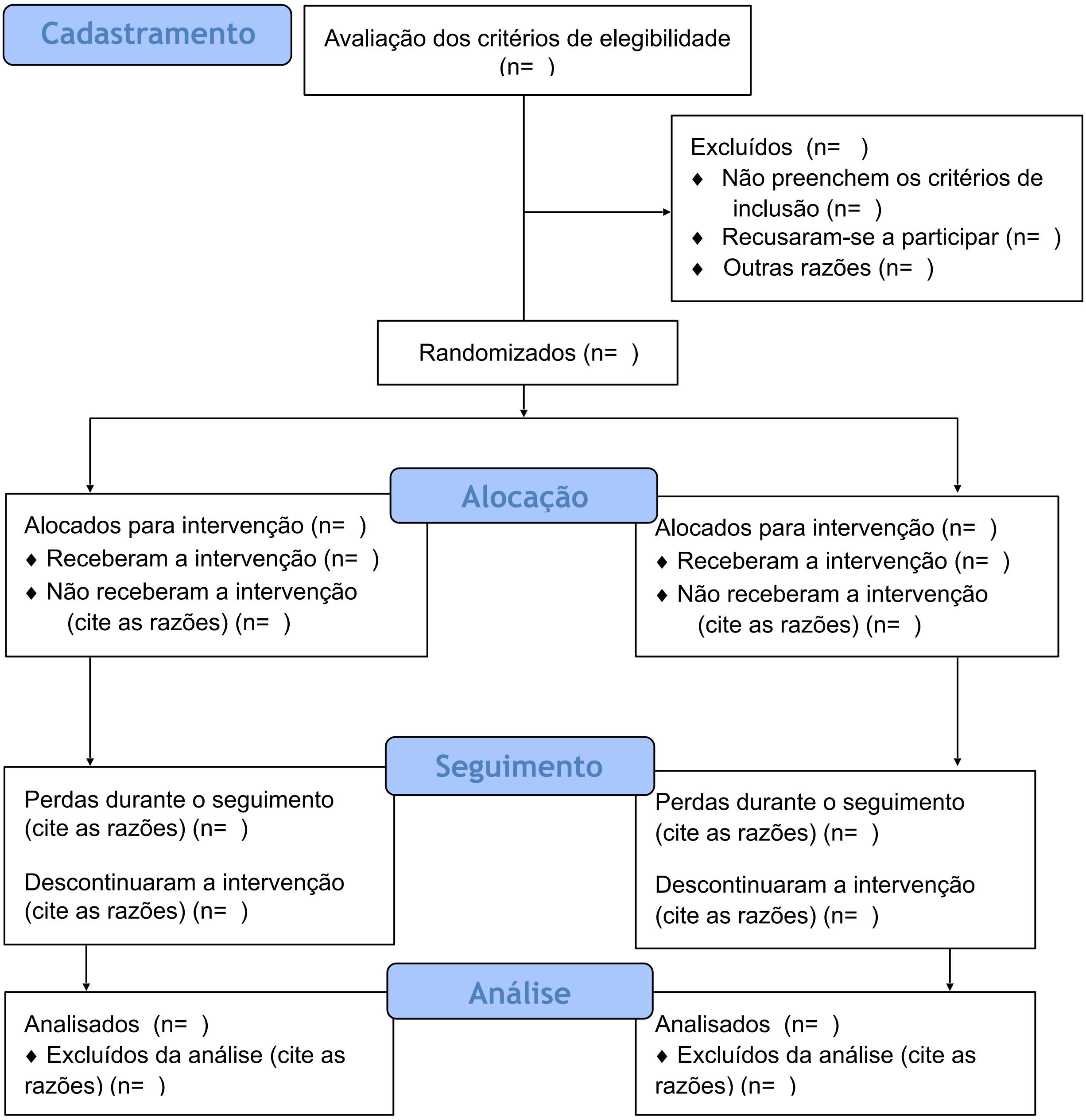

Figura 1. Fluxograma de captação e acompanhamento dos participantes

5.8. Termos, Variáveis e Conceitos

5.8.1. Variáveis de controle (para caracterização da amostra)

- Idade
- Peso
- Altura
- Sexo
- Estado físico segundo a ASA
- Tipo de cirurgia

5.8.2. Variável Independente

- Uso de CPAP

5.8.3. Variáveis Dependentes

- Tempo entre o início da apneia e a queda da saturação da oxihemoglobina a 95% (Tempo 1);
- Saturação de oxihemoglobina na oximetria de pulso durante a indução anestésica em diferentes momentos;
- Frequênçia de complicações: laringoespasmo, hipoxemia, bradicardia, parada cardiorrespiratória, morte;
- Tempo para recuperação dos níveis da saturação da oxihemoglobina na oximetria de pulso pré-apneia (Tempo 2);

5.8.4. Definição de Termos e Variáveis

Termo/Variável	Definição	Categorização

CPAP	Modo ventilatório no qual o paciente respira espontaneamente através de um circuito pressurizado contra um resistor de limiar que mantém uma determinada pressão durante tanto na inspiração quanto na expiração[31]	Variável independente
Apneia central	Cessação transitória da respiração por qualquer duração usualmente acompanhada de bradicardia e/ou cianose[32]	-
Ventilação pulmonar	Volume total de gás inspirado ou expirado na unidade de tempo[33]	-
Pré-escolar	Criança cuja faixa etária vai dos dois anos completos aos seis anos incompletos	-

Oxihemoglobina	Composto formado pela combinação de hemoglobina e oxigênio no qual este último se liga diretamente ao ferro sem causar mudança deste do estado ferroso para o férrico	-
Oximetria de pulso	Determinação da saturação da oxihemoglobina do sangue através de eletrodos ligados a alguma parte translucente do corpo (dedo, lobo da orelha, dobra da pele)[34]	-
Laringoespasmo	Complicação respiratória decorrente do fechamento reflexo da glote mais comum em crianças durante anestesia superficial[35]	Variável dependente, qualitativa, nominal, dicotômica, tendo como categorias sim ou não

Anestesia geral	Estágio transitório de inconsciência induzido por drogas através de suas ações em receptores moleculares[1]	-
Doença pulmonar paren-quimatosa	Grupo diverso de doenças pulmonares caracterizado inicialmente por inflama-ção dos alvéolos que se extende para o interstício, levando a fibrose pulmonar difusa	-
Cianose	Coloração azulada ou púr-pura da pele e mucosas de-vido a aumento da hemo-globina desoxigenada no sangue ou por um defeito estrutural da molécula de hemoglobina	-

Infecção do trato respiratório superior	Invasão do trato respiratório superior do hospedeiro por um patógeno, normalmente vírus ou bactéria. As principais doenças classificadas nesse grupo são rinofaringite viral, sinusite aguda, faringoamidalite aguda estreptocócica e laringite viral aguda. A rinofaringite viral aguda é a representante mais comum desse grupo e é caracterizada por dor de garganta, coriza, obstrução nasal, espirros, tosse seca e febre de intensidade variável[36]	-

Tempo entre o início da apneia e a queda da saturação da oxihemoglobina a 95%	-	Variável dependente, quantitativa, numérica, contínua, medida em segundos
Saturação de oxihemoglobina na oximetria de pulso durante a indução anestésica em diferentes momentos	-	Variável dependente, quantitativa, numérica, discreta, medida em pontos percentuais
Tempo para recuperação dos níveis da saturação da oxihemoglobina na oximetria de pulso pré-apneia	-	Variável dependente, quantitativa, numérica, contínua, medida em segundos
Idade	-	Variável quantitativa, numérica, contínua, medida em meses completos de vida
Peso	-	Variável quantitativa, numérica, contínua, medida em gramas
Altura	-	Variável quantitativa, numérica, contínua, medida em centímetros

Sexo	-	Variável qualitativa, nominal, dicotômica, tendo como categorias masculino e feminino
Estado físico segundo a Sociedade Americana de Anestesiologia (ASA)	Estado físico do paciente baseado na presença/ausência de doença nos pacientes ou em alterações fisiológicas não patológicas, bem como na qualidade do controle das morbidades[37]	Variável ordinal cujas categorias são numeradas em algarismos romanos de I a VI, sendo os estados I, ausência de doença sistêmica, e II, doença sistêmica controlada[38]

5.9. Procedimentos, testes, técnicas e exames

5.9.1. Realização da anestesia:

Os pacientes selecionados para o estudo serão admitidos na sala de operação e receberão monitorização habitual (cardioscópio, oxímetro, pressão arterial não invasiva e capnografia).

A indução inalatória será com sevoflurano a 8%, fração inspirada de oxigênio de 60,5% sob um fluxo de gases frescos de 4l/min (2l de oxigênio e 2l de ar comprimido) até perda do reflexo palpebral. A concentração do anestésico será então reduzida para 4%. Será utilizada máscara facial acoplada ao aparelho de anestesia, fixada ao paciente através de faixa elástica.

Após a adequada ventilação ser constatada através do correto posicionamento da máscara facial e de curva de capnografia presente, será obtido acesso venoso periférico com cateter venoso número 20, 22 ou 24G para hidratação e infusão de propofol na dose de 3,5mg/kg para induzir apneia nos pacientes de ambos os grupos.

5.9.2. Realização do CPAP

Os pacientes serão submetidos logo após a monitorização à técnica descrita nos envelopes entregues na entrada do bloco cirúrgico.

No grupo CPAP, este será aplicado no aparelho de anestesia (Dräger Fabius GS), utilizando um sistema circular. Esse sistema consiste de dois tubos corrugados acoplados numa extremidade a uma peça em Y, conectada à máscara facial do paciente, e na outra extremidade ao aparelho de anestesia que, além de fornecer o fluxo de gases frescos, possui um absorvedor de gás carbônico chamado cal sodada. Essa última além de possibilitar o sistema ser circular por retirar o CO_2 do ar fornecido ao paciente, aquece e umidifica a mistura gasosa. Uma válvula limitadora de pressão (válvula pop-off), que impede a perda de gases pelo sistema paciente-aparelho quando fechada, é parte integrante do aparelho de anestesia. Aquela possui diversas marcações (0-70cmH_2O), é manipulada manualmente e pode estar aberta (0cmH_2O), ou seja, sem que nenhuma pressão seja fornecida à via aérea do paciente, ou fechada. Na posição fechada, haverá uma pressão positiva contínua sendo fornecida à via aérea do paciente. A pressão que será utilizada neste grupo será de 10cmH_2O.

No grupo Sistema Aberto, o sistema permanecerá com a válvula na posição aberta, ou seja, 0cmH_2O.

Em ambos os grupos, os pacientes ventilarão espontaneamente desde o princípio com a técnica definida no momento da alocação.

Após o início da apnéia, o tempo será cronometrado e, periodicamente (a cada 10 segundos), será registrada a saturação da oxihemoglobina através do oxímetro de pulso por um limite de tempo máximo de cinco minutos afim de flagrar uma saturação mínima de 95% em ambos os grupos.

Todo o procedimento anestésico terá a presença do anestesista que acompanha o paciente e ficará a critério daquele assim que o estudo terminar.

5.10. Procedimentos para coleta dos dados

5.10.1. Instrumento de coleta de dados

Os dados serão coletados utilizando um formulário padronizado, pré-codificado para entrada dos dados no computador (Apêndice 3). As informações das variáveis categóricas serão pré-codificadas e as variáveis contínuas expressas em seu próprio valor numérico e só no momento da análise os resultados de algumas destas serão categorizados.

Esses formulários serão devidamente armazenados em pastas de arquivo específicos, antes e depois da digitação e análise, sob responsabilidade do próprio pesquisador, que os preencherá em diferentes momentos, antes, durante e após o procedimento cirúrgico.

5.10.2. Coleta de dados

Os dados serão coletados por um pesquisador independente que estará presente na sala de cirurgia, preencherá o formulário com os dados de identificação do paciente e as variáveis de estudo e não interferirá no procedimento anestésico que deverá ser

realizado.

O tempo a partir do momento da cessação dos movimentos respiratórios e decaimento da curva de capnografia será cronometrado. A medição acontecerá até o registro na oximetria de pulso do valor de 95%. Ventilação assistida será então instituída (no grupo Sistema Aberto, a válvula pop-off será fechada manualmente até o valor de 10cmH_2O). Haverá nesse momento nova cronometragem do tempo até leitura na oximetria de pulso do valor de 100% ou do valor obtido imediatamente antes do início da apneia.

5.11. Processamento e análise dos dados

5.11.1. Processamento dos dados

A digitação no banco de dados específico criado no programa excel será realizada duas vezes, em épocas e por pessoas diferentes, obtendo-se ao final uma listagem para correção de eventuais erros de digitação, com supervisão do próprio pesquisador.

Em se constatando inconsistências ou ausência de dados por ocasião da revisão das listagens, serão consultados os formulários arquivados correspondentes, de acordo com o número de registro dos pacientes.

Ao término da entrada de todos os formulários no banco de dados, será realizada a revisão final e completando-se os dados ausentes pelos processos acima mencionados. O banco de dados definitivo assim criado será, então, utilizado para análise estatística no programa STATA, sendo ainda submetido a testes de consistência e limpeza das informações, gerando-se cópias de segurança.

5.11.2. Análise dos dados

A análise dos dados será realizada pela pesquisadora e pelo estatístico responsável (supervisionada por sua orientadora), utilizando o programa STATA.

A análise estatística descritiva será realizada através de medidas de tendência central e de dispersão para as variáveis quantitativas e através distribuição de frequências para as variáveis qualitativas. Para comparação de amostras pareadas, será utilizado um método não-paramétrico, teste T de Wilcoxcon.

Será utilizado teste t de Student para duas amostras independentes considerando previamente o teste de normalidade de Kolgomorov-Smirnoff para cada amostra, e para o caso de não normalidade será usado o teste não paramétrico U de Mann-Whitney. As variáveis quantitativas segundo o tempo de sua realização serão utilizadas análise de variância para as unidades repetidas (ANOVA), se apresentarem uma distribuição normal, ou Wilcoxcon caso não esteja presente a normalidade. Para as variáveis categóricas será utilizado o teste exato de Fisher ou Qui-quadrado, quando necessário.

Será ainda calculado o Risco Relativo e seu intervalo de confiança a 95%, além do Número Necessário para Tratar e obter um benefício (NNT) ou o Número Necessário para Tratar ou se obter um malefício (NNH) quando pertinente.

5.12. Aspectos éticos

A pesquisa respeitará os direitos humanos e os princípios da bioética (Autonomia, Não-Maleficência, Beneficência, Justiça e Eqüidade). O sigilo e a confidencialidade na coleta e arquivo dos dados colhidos serão respeitados.

Os termos da resolução nº 466 de 12 de dezembro de 2012 do Conselho Nacional de Saúde para pesquisa em seres humanos serão seguidos assim como a

declaração de Helsinque. Além disso, o projeto será submetido à apreciação do Comitê de Ética em Pesquisa da instituição proponente, os dados somente serão colhidos após tal submissão e aprovação do referido comitê e as crianças do estudo somente serão incluídas após os responsáveis assinarem o Termo de Consentimento Livre e Esclarecido (TCLE, Apêndice 2).

O estudo será registrado no *ClinicalTrials.gov* e não oferecerá riscos ou desconfortos adicionais, além daqueles inerentes ao próprio procedimento anestésico-cirúrgico, assim como também não foram relatados na literatura efeitos adversos que contraindiquem o uso de CPAP durante a indução. Ao contrário, os poucos estudos existentes referem melhora respiratória com o uso desta intervenção. Todos os procedimentos da pesquisa serão realizados por profissionais treinados e capacitados, tanto para a realização da anestesia pediátrica, como para a realização do CPAP.

O TCLE (apêndice 2) participará aos responsáveis todas as informações com relação às vantagens e às desvantagens do uso das duas técnicas; enfatizará que não será realizado nenhum procedimento já não utilizada de rotina pela equipe; e nele constará o direito de se recusarem a participar do estudo, bem como a garantia de assistência àqueles que não aceitarem, sem haver ressarcimento por parte da instituição, nem por parte dos pesquisadores. Estes se comprometerão a publicar o estudo, independentemente dos resultados obtidos.

5.13. Conflitos de interesse

Esta pesquisa estará livre de conflito de interesses, particular ou institucional. Não haverá financiamento por parte de indústria farmacêutica ou de representantes de nenhum objeto de pesquisa utilizado.

VI. PLANO DE RESULTADOS

Tabela 1. Variáveis para caracterização da amostra de pré-escolares submetidos a anestesia geral para cirurgia pediátrica eletiva no HC-UFPE em 2018

Variável	Grupo C (n)	Grupo A (n)
**Idade, meses (média ± DP)**		
**Peso, gramas (média ± DP)**		
**Altura, cm (média ± DP)**		
**Sexo (n/%)**		
Masculino		
Feminino		
**ASA (n/%)**		
I		
II		
**Cirurgia pediátrica (n/%)**		
Postectomia		
Herniorrafia umbilical		
Herniorrafia inguinal		
Correção de hipospádia		
Orquidopexia		

DP - desvio padrão, Grupo C - grupo CPAP, Grupo A - grupo sistema aberto, ASA - estado físico segundo a Sociedade Americana de Anestesiologia

Tabela 2. Tempos entre o início da apneia e saturação de 95%, e para recuperação dos níveis de saturação pré-apneia de pré-escolares submetidos a anestesia geral para cirurgia pediátrica eletiva no HC-UFPE em 2018

**Variável**	**Grupo C (n)**	**Grupo A (n)**	**Range**	**IC95%**	*p*
**Tempo (em segundos) entre o início da apneia e saturação de 95% (média ± DP)**					*
**Tempo (em segundos) para recuperação dos níveis de saturação pré-apneia (média ± DP)**					*

* teste *t* de Student, DP - desvio padrão, Grupo C - grupo CPAP, Grupo A - grupo sistema aberto

Tabela 3. Saturação de oxigênio durante a indução anestésica em diferentes momentos de pré-escolares submetidos a anestesia geral para cirurgia pediátrica eletiva no HC-UFPE em 2018

**Variável**	**Grupo C (n)**	**Grupo A (n)**	**Range**	**IC95%**	*p*
**Saturação de oxigênio durante a indução anestésica (média ± DP)**					*
10”					
20”					
30”					
40”					
50”					
60”					
70”					
80”					
90”					
100”					
110”					
120”					
130”					
140”					
150”					

* teste *t* de Student, DP - desvio padrão, Grupo C - grupo CPAP, Grupo A - grupo sistema aberto

Tabela 4. Frequências de complicações durante a indução em pré-escolares submetidos a anestesia geral para cirurgia pediátrica eletiva no HC-UFPE em 2018

**Variável**	**Grupo C (n)**	**Grupo A (n)**	**RR**	**IC95%**	*p*
**Complicações (n/%)**					*
Laringoes-pasmo					**
Hipoxemia					**
Bradicardia					**
Parada cardía-ca					**
Morte					**

* teste z, ** teste X^2 de Pearson, DP - desvio padrão, Grupo C - grupo CPAP, Grupo A - grupo sistema aberto

VII. ORÇAMENTO

Os gastos ficarão por conta dos autores do trabalho. As medicações utilizadas assim como materiais para anestesia são de uso corrente no Serviço de Anestesiologia para realização de procedimentos de rotina no bloco cirúrgico do HC-UFPE.

ITEM	RECURSO HUMANO/ MATERIAL	PREÇO UNITÁRIO (R$)	PREÇO TOTAL (R$)
01	Professor (Coordenador do serviço)	Sem custo	0
01	Estatístico	R$2.000, 00	R$2.000, 00
500	Papel sulfite branco, 75g/m², A4	R$21,90	R$21,90
03	Cartuchos da impressora Epson XP-401 preto	R$30,00	R$90,00
200	Fotocópias	R$0,20	R$40,00
01	Materiais de Escritório (canetas, pranchetas, marca-texto, grampeador, grampos)	R$30,00	R$30,00
01	Revisor de texto	R$ 350,00	R$ 350,00
01	Tradutor	R$ 3.000,00	R$ 3.000,00
01	Taxa de publicação	R$ 5.000,00	R$ 5.000,00
TOTAL			R$10.501,90

____ Pesquisador

VIII. CRONOGRAMA

	Set/17	Out/17	Nov/17	Dez/17	Jan/18	Fev/18	Mar/18	Abr/18	Mai/18	Jun/18	Jul/18	Ago/18	Set/18	Out/18	Nov/18	Dez/18	Jan/19	Fev/19
Elaboração do projeto	X																	
Revisão da literatura	X	X	X	X	X	X	X	X	X	X	X	X	X	X	X	X	X	
Apresentação do projeto		X																
Preparação da equipe e teste do instrumento		X	X															
Coleta dos dados				X	X	X	X	X	X	X								
Revisão e correção dos formulários										X								
Digitação											X							
Limpeza e testes de consistência												X						
Tabulação e análise dos dados													X					
Revisão da análise dos dados													X					
Redação da dissertação													X	X				
Revisão da dissertação														X				
Redação do artigo															X	X	X	
Defesa da Dissertação																		X
Entrega do artigo																		X

A coleta de dados só será iniciada após a aprovação do projeto pelo Comitê de Ética em Pesquisa (CEP).

IX. REFERÊNCIAS

1. Århem P, Klement G, Nilsson J. Mechanisms of Anesthesia: Towards Integrating Network, Cellular, and Molecular Level Modeling. *Neuropsychopharmacology*. 2003;28(S1):S40-S47.
2. Schwartz RS, Brown EN, Lydic R, Schiff ND. General Anesthesia, Sleep, and Coma. *N Engl J Med*. 2010;363(27):2638-2650.
3. Boonmak P, Boonmak S, Pattanittum P. High initial concentration versus low initial concentration sevoflurane for inhalational induction of anaesthesia. Boonmak P, ed. *Cochrane database Syst Rev*. 2012;2016(9):CD006837.
4. Park JH, Kim JY, Lee JM, Kim YH, Jeong HW, Kil HK. Manual vs. pressure-controlled facemask ventilation for anaesthetic induction in paralysed children: a randomised controlled trial. *Acta Anaesthesiol Scand*. 2016;60(8):1075-1083.
5. von Ungern-Sternberg BS, Boda K, Chambers NA, et al. Risk assessment for respiratory complications in paediatric anaesthesia: a prospective cohort study. *Lancet*. 2010;376(9743):773-783.
6. Ehsan Z, Mahmoud M, Shott SR, Amin RS, Ishman SL. The effects of Anesthesia and opioids on the upper airway: A systematic review. *Laryngoscope*. 2016;126(1):270-284.
7. Hedenstierna G, Edmark L. Effects of anesthesia on the respiratory system. *Best Pract Res Clin Anaesthesiol*. 2015;29(3):273-284.
8. Bouroche G, Bourgain JL. Preoxygenation and general anesthesia: a review. *Minerva Anestesiol*. 2015;81(8):910-920.

9. Nimmagadda U, Salem MR, Crystal GJ. Preoxygenation: Physiologic Basis, Benefits, and Potential Risks. *Anesth Analg*. 2017;124(2):507-517.

10. Gonzalez L, Pignaton W, Kusano P, Modolo N, Braz J, Braz L. Anesthesia-related mortality in pediatric patients: a systematic review. *Clinics*. 2012;67(4):381-387.

11. Fiadjoe JE, Nishisaki A, Jagannathan N, et al. Airway management complications in children with difficult tracheal intubation from the Pediatric Difficult Intubation (PeDI) registry: a prospective cohort analysis. *Lancet Respir Med*. 2016;4(1):37-48.

12. Long E, Sabato S, Babl FE. Endotracheal intubation in the pediatric emergency department. Anderson B, ed. *Pediatr Anesth*. 2014;24(12):1204-1211.

13. King MR, Anderson TA, Sui J, He G, Poon KYT, Coté CJ. Age-related incidence of desaturation events and the cardiac responses on stroke index, cardiac index, and heart rate measured by continuous bioimpedance noninvasive cardiac output monitoring in infants and children undergoing general anesthesia. *J Clin Anesth*. 2016;32:181-188.

14. Bharti N, Batra YK, Kaur H. Paediatric perioperative cardiac arrest and its mortality: database of a 60-month period from a tertiary care paediatric centre. *Eur J Anaesthesiol*. 2009;26(6):490-495.

15. Gonzalez LP, Braz JRC, Módolo MP, de Carvalho LR, Módolo NSP, Braz LG. Pediatric Perioperative Cardiac Arrest and Mortality. *Pediatr Crit Care Med*. 2014;15(9):878-884.

16. Bhananker S, Harless J, Ramaiah R. Pediatric airway management. *Int J Crit Illn Inj Sci*. 2014;4(1):65.

17. Chiron B, Mas C, Ferrandière M, et al. Standard preoxygenation vs two techniques in children. *Paediatr Anaesth*. 2007;17(10):963-967.

18. De Jong A, Futier E, Millot A, et al. How to preoxygenate in operative room: healthy subjects and situations "at risk". *Ann Fr Anesth Reanim*. 2014;33(7-8):457-461.

19. Humphreys S, Lee-Archer P, Reyne G, Long D, Williams T, Schibler A. Transnasal humidified rapid-insufflation ventilatory exchange (THRIVE) in children: a randomized controlled trial. *Br J Anaesth*. 2017;118(2):232-238.

20. Windpassinger M, Plattner O, Gemeiner J, et al. Pharyngeal Oxygen Insufflation During AirTraq Laryngoscopy Slows Arterial Desaturation in Infants and Small Children. *Anesth Analg*. 2016;122(4):1153-1157.

21. Faria DAS, da Silva EMK, Atallah ÁN, Vital FMR. Noninvasive positive pressure ventilation for acute respiratory failure following upper abdominal surgery. Vital FM, ed. *Cochrane database Syst Rev*. 2015;(10):CD009134.

22. Bratton DJ, Stradling JR, Barbé F, Kohler M. Effect of CPAP on blood pressure in patients with minimally symptomatic obstructive sleep apnoea: a meta-analysis using individual patient data from four randomised controlled trials. *Thorax*. 2014;69(12):1128-1135.

23. Wang J, Yu W, Gao M, et al. Continuous positive airway pressure treatment reduces cardiovascular death and non-fatal cardiovascular events in patients with

obstructive sleep apnea: A meta-analysis of 11 studies. *Int J Cardiol*. 2015;191:128-131.

24. Iftikhar IH, Khan MF, Das A, Magalang UJ. Meta-analysis: Continuous Positive Airway Pressure Improves Insulin Resistance in Patients with Sleep Apnea without Diabetes. *Ann Am Thorac Soc*. 2013;10(2):115-120.
25. de Freitas Dantas Gomes EL, Costa D, Germano SM, Borges PV, Sampaio LMM. Effects of CPAP on clinical variables and autonomic modulation in children during an asthma attack. *Respir Physiol Neurobiol*. 2013;188(1):66-70.
26. Jat KR, Mathew JL. Continuous positive airway pressure (CPAP) for acute bronchiolitis in children. Jat KR, ed. *Cochrane database Syst Rev*. 2015;1:CD010473.
27. Sinha IP, McBride AKS, Smith R, Fernandes RM. CPAP and High-Flow Nasal Cannula Oxygen in Bronchiolitis. *Chest*. 2015;148(3):810-823.
28. Wilson PT, Morris MC, Biagas K V., Otupiri E, Moresky RT. A randomized clinical trial evaluating nasal continuous positive airway pressure for acute respiratory distress in a developing country. *J Pediatr*. 2013;162(5):988-992.
29. Sreejit M, Ramkumar V. Effect of positive airway pressure during pre-oxygenation and induction of anaesthesia upon safe duration of apnoea. *Indian J Anaesth*. 2015;59(4):216.
30. Harbut P, Gozdzik W, Stjernfält E, Marsk R, Hesselvik JF. Continuous positive airway pressure/pressure support pre-oxygenation of morbidly obese patients. *Acta Anaesthesiol Scand*. 2014;58(6):675-680.

31. Squadrone V, Coha M, Cerutti E, et al. Continuous positive airway pressure for treatment of postoperative hypoxemia: a randomized controlled trial. *JAMA*. 2005;293(5):589-595.

32. Pradhapan P, Swaminathan M, Salila Vijayalal Mohan HK, Sriraam N. Identification of apnea during respiratory monitoring using support vector machine classifier: a pilot study. *J Clin Monit Comput*. 2013;27(2):179-185.

33. Simon BA, Kaczka DW, Bankier AA, Parraga G. What can computed tomography and magnetic resonance imaging tell us about ventilation? *J Appl Physiol*. 2012;113(4):647-657.

34. Jubran A. Pulse oximetry. *Crit Care*. 2015;19(1):272.

35. Roy WL, Lerman J. Laryngospasm in paediatric anaesthesia. *Can J Anaesth*. 1988;35(1):93-98.

36. Pitrez PMC, Pitrez JLB. Acute upper respiratory tract infections: outpatient diagnosis and treatment. *J Pediatr* (Rio J). 2003;79(suppl.1):77-86. http://dx.doi.org/10.1590/S0021-75572003000700009.

37. Saklad M. Grading of patients for surgical procedures. *Anesthesiology*. 1941;2(3):281-284.

38. ASA Physical Status Classification System. https://www.asahq.org/resources/clinical-information/asa-physical-status-classification-system. Published 2014.

APÊNDICE 1 - LISTA DE CHECAGEM

DATA

Nome: ……………………………………………………………………………………….

CRITÉRIOS DE INCLUSÃO

	Sim	Não
ASA I ou II		
Crianças em idade pré-escolar		
Crianças submetidas a anestesia geral para cirurgia pediátrica eletiva		

CRITÉRIOS DE EXCLUSÃO

	Sim	Não
Doença pulmonar parenquimatosa pré-existente		
Crianças cianóticas ou com saturação da oxihemoglobina menor que 95% antes da indução anestésica		
História recente (<4 semanas) ou vigência de infecção do trato respiratório superior		

Elegível: Sim ( ) Não ( )

Responsável assinou termo de consentimento livre e esclarecido: Sim ( ) Não ( )

APÊNDICE 2 - TERMO DE CONSENTIMENTO LIVRE E ESCLARECIDO

Instituto de Medicina Integral Prof. Fernando Figueira/Escola de Pós-graduação em Saúde Materno Infantil

TERMO DE CONSENTIMENTO LIVRE E ESCLARECIDO

Solicitamos a sua autorização para convidar o (a) seu/sua filho (a)________________ {ou menor que está sob sua responsabilidade} para participar, como voluntário (a), da pesquisa PRESSÃO POSITIVA CONTÍNUA NAS VIAS AÉREAS DURANTE A INDUÇÃO DE ANESTESIA GERAL PARA CIRURGIA PEDIÁTRICA ELETIVA: ENSAIO CLÍNICO RANDOMIZADO. Para que você possa decidir se quer ou não participar, precisa conhecer os benefícios, riscos e consequências da sua participação.

Este documento é chamado de Termo de Consentimento Livre e Esclarecido (TCLE) e tem esse nome pois você só deve participar da pesquisa depois de ter lido e entendido essa declaração. Leia as informações com atenção e converse com o pesquisador responsável e equipe da pesquisa sobre quaisquer dúvidas que você tenha. Caso haja uma palavra ou termo que você não entenda, converse com a pessoa responsável por obter esse consentimento, para maiores esclarecimentos. Caso prefira, converse com seus familiares, amigos e equipe médica antes de tomar uma decisão. Após receber todas as informações, você poderá fornecer seu consentimento, rubricando e/ou assinando as duas vias, uma do pesquisador responsável e outra do participante da pesquisa.

Caso não concorde, não haverá penalização nem para o (a) Sr.(a) nem para a criança que está sob sua responsabilidade, bem como será possível ao/a Sr. (a) retirar o consentimento a qualquer momento, também sem nenhuma penalidade.

Trata-se de uma pesquisa, que é da responsabilidade do pesquisador Jayme Marques dos Santos Neto, que é anestesista e esta estudando uma técnica para ajudar na respiração das crianças durante a cirurgia. O endereço do pesquisador é : Avenida Boa Viagem, 306 apto. 701, Pina, Recife-PE CEP 51011-000; telefone (81)996212977. Dr. Jayme está fazendo a dissertação de Mestrado e esta sendo orientado pela também Anestesista Dra. Flávia Augusta de Orange Lins da Fonseca e Silva, Telefone 81994197979, e-mail orangeflavia@gmail.com

INFORMAÇÕES SOBRE A PESQUISA:

Sua criança está sendo convidada a ser participante de uma pesquisa que estudará o efeito de uma técnica para ajudar na respiração durante o início da anestesia. Chamamos esta técnica de pressão positiva contínua (CPAP).

Para a criança ser operada, ela precisa receber anestesia geral. Para isso, ela respira usando uma máscara de silicone ou plástico que está conectada ao aparelho de anestesia. O gás que vem do aparelho que faz anestesia contém o remédio que faz a criança dormir. Essa técnica é conhecida popularmente como “cheirinho”, mas na verdade é Anestesia Geral, e é usada de forma corriqueira em praticamente todas as anestesias em crianças. O CPAP é feito dessa mesma maneira. A única diferença para a ventilação habitual é que no CPAP o aparelho de anestesia fornece uma pressão que pode ajudar (é o que queremos descobrir) a respirar melhor.

Serão formados dois grupos em que num deles as crianças receberão o CPAP e no outro as crianças receberão a ventilação habitual. Não sabemos em qual grupo sua criança ficará. A participação dela não é obrigatória. O objetivo deste projeto é saber se

o CPAP no início da anestesia melhora a segurança da criança e se o oxigênio no sangue dela permanece em níveis normais por mais tempo.

A participação da sua criança termina tão logo ela volte a condição inicial no estudo quando a sua cirurgia será então realizada. Queremos deixar claro não se tratar de método novo, que já vem sendo utilizado, sendo considerada técnica segura. Em estudos anteriores, não foram verificados efeitos colaterais ou complicações.

Espera-se que, como resultado deste estudo, possa ser estimulado cada vez mais o uso de CPAP no início da anestesia, melhorando a qualidade da assistência na anestesia. Toda a anestesia terá a participação do médico anestesiologista responsável pela cirurgia além do pesquisador que estará presente durante todo o período do estudo, aumentando assim a vigilância sobre os procedimentos realizados na sua criança.

Efeitos indesejáveis são possíveis de ocorrer em qualquer estudo de pesquisa, tais como constrangimento ao assinar este termo, apesar de todos os cuidados possíveis, e podem acontecer sem que a culpa seja sua ou dos pesquisadores. Se sua criança sofrer efeitos indesejáveis como dano associado da sua participação neste estudo, a assistência imediata e integral profissional será providenciada.

As possíveis vantagens para sua criança são maior quantidade de oxigênio no sangue dela, menor chance de problemas no começo da anestesia, aumento do tempo de segurança para ela caso algum problema aconteça também no começo da anestesia e recuperação mais rápida se ela parar de respirar.

As informações desta pesquisa serão confidenciais e serão divulgadas apenas em eventos ou publicações científicas, não havendo identificação dos voluntários, a não ser entre os responsáveis pelo estudo, sendo assegurado o sigilo sobre a participação do/a

voluntário (a). Os dados coletados nesta pesquisa, através de formulários, ficarão armazenados em pastas de arquivo, sob a responsabilidade do pesquisador, no endereço acima informado, pelo período de mínimo 5 anos.

O (A) senhor(a) não pagará nada e nem receberá nenhum pagamento para ele/ela participar desta pesquisa, pois deve ser de forma voluntária, mas fica também garantida a indenização em casos de danos, comprovadamente decorrentes da participação dele/a na pesquisa, conforme decisão judicial ou extra-judicial. Se houver necessidade, as despesas para a participação serão assumidas pelos pesquisadores (ressarcimento com transporte e alimentação).

Em caso de dúvidas relacionadas aos aspectos éticos deste estudo, você poderá consultar o Comitê de Ética em Pesquisa Envolvendo Seres Humanos do IMIP no endereço: **Rua dos Coelhos, nº 300, Boa Vista. Diretoria de Pesquisa do IMIP, Prédio Administrativo Orlando Onofre, 1º Andar tel: 2122-4756 – Email: comitedeetica@imip.org.br**. O CEP/IMIP funciona de 2ª a 6ª feira, das 07:00 às 11:30h (manhã) e das 13:30 às 16:00h (tarde).

____

Assinatura do pesquisador (a)

CONSENTIMENTO DO RESPONSÁVEL PARA A PARTICIPAÇÃO DO/A VOLUNTÁRIO

Eu, ______________________________________, CPF__________________, abaixo assinado, responsável por __________________________________, autorizo a sua participação no estudo PRESSÃO POSITIVA CONTÍNUA NAS VIA AÉREAS

DURANTE A INDUÇÃO DE ANESTESIA GERAL PARA CIRURGIA PEDIÁTRICA ELETIVA: ENSAIO CLÍNICO RANDOMIZADO, como voluntário(a). Fui devidamente informado (a) e esclarecido (a) pelo (a) pesquisador (a) sobre a pesquisa, os procedimentos nela envolvidos, assim como os possíveis riscos e benefícios decorrentes da participação dele (a). Foi-me garantido que posso retirar o meu consentimento a qualquer momento, sem que isto leve a qualquer penalidade ou interrupção de seu acompanhamento/ assistência/tratamento para mim ou para o (a) menor em questão.

Local e data __________________

Assinatura do (da) responsável: __________________________

Presenciamos a solicitação de consentimento, esclarecimentos sobre a pesquisa e aceite do sujeito em participar. 02 testemunhas (não ligadas à equipe de pesquisadores):

Nome:	Nome:
Assinatura:	Assinatura:

APÊNDICE 3 – FORMULÁRIO

FORMULÁRIO Nº

GRUPO:

DATA

<u>Características do paciente</u>

Nome: ……………………………………………………………………………….

Data da admissão: / / ; Registro:

Data de nascimento : / / ; ASA: I II

Idade (meses): ; Peso (g): ; Altura (cm): ; Sexo: M ( ) F ( )

Cirurgia:

Valor da oximetria de pulso no início da apneia:

Tempo entre o início da apneia e a queda da saturação da oxihemoglobina a 95% (segundos):

Valores da oximetria de pulso

10”: ; 20”: ; 30”: ; 40”: ; 50”: ; 60”: ; 70”: ; 80”: ; 90”: ; 100”: ; 110”: ; 120”: ; 130”: ; 140”: ; 150”: .

Tempo para recuperação dos níveis da saturação da oxihemoglobina na oximetria de pulso pré-apneia (segundos):

Complicações: Laringoespasmo ( ) Hipoxemia ( ) Bradicardia ( ) Parada cardiorrespiratória ( ) Morte ( )
