# Supplementary material for: Continuous positive airway pressure to reduce the risk of early peripheral oxygen desaturation after onset of apnoea in children: A double-blind randomised controlled trial
Source: PLoS One. 2021 Oct 1;16(10):e0256950. doi: 10.1371/journal.pone.0256950 (PMC8486132; doi:10.1371/journal.pone.0256950)
Supplement: S3 File — (DOCX) [file pone.0256950.s006.docx]

INFORMED CONSENT FORM

We request your permission to invite your child ________________ (or minor under your responsibility) to volunteer for the survey CONTINUOUS POSITIVE PRESSURE ON THE AIRWAYS DURING THE INDUCTION OF GENERAL ANESTHESIA FOR ELECTIVE PEDIATRIC SURGERY: RANDOMIZED CLINICAL TEST. In order for you to decide whether or not to participate, you need to know the benefits, risks and consequences of your participation.

This document is called the Free and Informed Consent Term (TCLE) and has this name because you should only participate in the research after you have read and understood this statement. Read the information carefully and talk to the responsible researcher and research team about any questions you may have. If there is a word or a term you do not understand, talk to the person responsible for obtaining such consent for further clarification. If you prefer, talk to your family, friends and medical team before making a decision. After receiving all the information, you can provide your consent, initializing and / or signing the two tracks, one of the responsible researcher and another of the research participant.

If you do not agree, there will be no penalty for either the child or the child under your responsibility, and you will be able to withdraw your consent at any time, also without any penalty.

This is a research, which is the responsibility of the researcher Jayme Marques dos Santos Neto, who is an anesthesiologist and is studying a technique to help children breathe during surgery. The researcher's address is: Avenida Boa Viagem, 306 apto. 701, Pina, Recife-PE CEP 51011-000; telephone (81) 996212977. Dr. Jayme is doing the Master's dissertation and is being advised also by Anaesthesist Dr. Flavia Augusta of Orange Lins da Fonseca e Silva, Phone 81994197979, email orangeflavia@gmail.com

RESEARCH INFORMATION:

Your child is being invited to take part in research that will study the effect of a technique to aid breathing during the onset of anesthesia. We call this technique continuous positive pressure (CPAP).

For the child to undergo surgery, she needs to receive general anesthesia. For this, she breathes using a silicone or plastic mask that is connected to the anesthesia machine. The gas that comes from the device that does anesthesia contains the medicine that causes the child to sleep. This technique is popularly referred to as "sniffing," but is actually General Anesthesia, and is commonly used in virtually all anesthesia in children. CPAP is done in the same way. The only difference to the usual ventilation is that in CPAP the anesthesia device provides a pressure that can help (we want to find out) to breathe better.

Two groups will be formed in which one of the children will receive CPAP and the other the children will receive the usual ventilation. We do not know what group your child will be in. Her participation is not compulsory. The goal of this project is to know whether CPAP at the onset of anesthesia improves the child's safety and whether the oxygen in her blood remains at normal levels for longer.

Your child's participation ends as soon as she returns to the initial condition in the study when her surgery will then be performed. We want to make it clear that this is not a new method, which has already been used and is considered a safe technique. In previous studies, no side effects or complications were found.

It is expected that as a result of this study, the use of CPAP at the onset of anesthesia may be stimulated more and more, improving the quality of the anesthesia care. All anesthesia will have the participation of the anesthesiologist responsible for surgery plus the researcher who will be present throughout the study period, thus increasing the vigilance about the procedures performed on your child.

Undesirable effects are possible to occur in any research study, such as embarrassment in signing this term, despite all possible care, and can happen without the fault of yours or the researchers. If your child experiences undesirable side effects as an associated harm to your participation in this study, immediate and comprehensive professional care will be provided.

The possible advantages for your child are more oxygen in their blood, less chance of problems at the beginning of anesthesia, increased safety time for them if any problem happens also at the beginning of anesthesia and faster recovery if they stop breathing.

The information of this research will be confidential and will only be divulged in scientific events or publications, without the identification of the volunteers, other than those in charge of the study, being kept confidential about the participation of the volunteer. The data collected in this research, through forms, will be stored in archives under the responsibility of the researcher at the above address for a period of at least 5 years. You will not pay anything and will not receive any payment for him / her to participate of this research, since it must be voluntary, but also indemnity is guaranteed in cases of damages, evidently arising from his participation in the research, according to judicial or extrajudicial decision. If necessary, the expenses for participation will be borne by the researchers (reimbursement with transportation and food). In case of doubts related to the ethical aspects of this study, you can consult the Research Ethics Committee Involving Human Beings of IMIP at Rua dos Coelhos, nº 300, Boa Vista. IMIP Research Directorate, Orlando Onofre Administrative Building, 1st Floor tel: 2122-4756 - Email: comitedeetica@imip.org.br. CEP / IMIP is open from Monday to Friday, from 7:00 am to 11:30 am and from 1:30 to 4:00 pm (afternoon) .________________________________________________________________ Signature of the researcher (a) CONSENT OF THE PERSON RESPONSIBLE FOR THE PARTICIPATION OF THE A VOLUNTÁRIOEu, _____________________________________, CPF_________________, undersigned, responsible for _______________________________, authorize their participation in the study CONTINUOUS POSITIVE PRESSURE ON THE AIRWAY DURING THE INDUCTION OF GENERAL ANESTHESIA FOR ELECTIVE PEDIATRIC SURGERY: RANDOMIZED CLINICAL TEST, as a volunteer. I was duly informed and informed by the researcher about the research, the procedures involved, as well as the possible risks and benefits arising from his / her participation. I have been guaranteed that I can withdraw my consent at any time, without this leading to any penalty or interruption of your follow-up / care / treatment for myself or the child in question

Protocol

METHODS5.1. Study design This is a randomized, phase III, parallel trial in pediatric patients submitted to elective surgeries.5.2. Place of studyThe study will be developed in the surgical block of the Hospital das Clínicas of the Federal University of Pernambuco (HC-UFPE). The pediatric surgery service of the HC-UFPE performs about 15-20 surgeries per week in an elective; the team is composed of six surgeons and three resident physicians; and has a room in the surgical ward in four weekly shifts in which he performs procedures on children from birth to age 18.5.3. Study period The study will be carried out from January 2018 to June 2018.5.4. Study population Pre-school pediatric patients undergoing elective surgery in the surgical ward of the Hospital das Clínicas.5.5. Sample5.5.1. Sampling A non-probabilistic convenience sample composed of pre-school children who will undergo general anesthesia for elective surgeries will be obtained, obeying the inclusion and exclusion criteria of the study.5.5.2. Sample Size Sample size calculation was performed on the Openepi program, version 3.01 (Dean AG, Sullivan KM, Soc. MM. OpenEpi: Open Source Epidemiologic Statistics for Public Health, Version www.OpenEpi.com, updated 2013/04/06 , accessed 2017/07/11), using averages difference. The first parameter used was the mean time in apnea that the patients exposed to the intervention led to an oxygen saturation in pulse oximetry of 95% (166 ± 47 seconds) 18. The second parameter was the mean time in apnea that patients not exposed to the intervention led to an oxygen saturation in 95% pulse oximetry (131 ± 39 seconds) 18. Considering a level of significance of 5% and a power of 90%, it will require 64 patients (32 in each group). However, predicting eventual post-randomization exclusion losses (around 10%), this number will be increased to 72 (36 in each group) .5.5.3. Procedure for randomization The randomization table will be generated on the computer using the Random Software Allocation program. After randomization, opaque envelopes sequentially numbered from 1 to 72 will be prepared according to the random number table. Allocation concealment will be respected.5.6. Criteria and procedures for selecting, capturing and monitoring participants5.6.1. Inclusion criteriaChildren of pre-school age ASA I or II Children undergoing general anesthesia for elective surgery 5.6.2. Exclusion criteria: Pre-existing parenchymal pulmonary disease; Cyanotic children with oxyhemoglobin saturation <95% prior to anesthetic induction; Recent history (<4 weeks) or duration of upper respiratory tract infection; Procedures for Capture and Follow-up of Participants The captation of participants will be carried out by a student of scientific initiation who will not participate in the collection of data. It will be solely responsible for recruiting participants, applying the eligibility criteria using a checklist (Appendix 1) and requesting the signature of the TCLE. This step will happen at the entrance of the surgical block where the patients and companions, coming from the ambulatory after weighing and measuring of the study. The study will be developed in the surgical block of the Hospital das Clínicas of the Federal University of Pernambuco (HC-UFPE). The pediatric surgery service of the HC-UFPE performs about 15-20 surgeries per week in an elective; the team is composed of six surgeons and three resident physicians; and has a room in the surgical ward in four weekly shifts in which he performs procedures on children from birth to age 18.5.3. Study period The study will be carried out from January 2018 to June 2018.5.4. Study population Pre-school pediatric patients undergoing elective surgery in the surgical ward of the Hospital das Clínicas.5.5. Sample5.5.1. Sampling A non-probabilistic convenience sample composed of pre-school children who will undergo general anesthesia for elective surgeries will be obtained, obeying the inclusion and exclusion criteria of the study.5.5.2. Sample Size Sample size calculation was performed on the Openepi program, version 3.01 (Dean AG, Sullivan KM, Soc. MM. OpenEpi: Open Source Epidemiologic Statistics for Public Health, Version www.OpenEpi.com, updated 2013/04/06 , accessed 2017/07/11), using averages difference. The first parameter used was the mean time in apnea that the patients exposed to the intervention led to an oxygen saturation in pulse oximetry of 95% (166 ± 47 seconds) 18. The second parameter was the mean time in apnea that patients not exposed to the intervention led to an oxygen saturation in 95% pulse oximetry (131 ± 39 seconds) 18. Considering a level of significance of 5% and a power of 90%, it will require 64 patients (32 in each group). However, predicting eventual post-randomization exclusion losses (around 10%), this number will be increased to 72 (36 in each group) .5.5.3. Procedure for randomization The randomization table will be generated on the computer using the Random Software Allocation program. After randomization, opaque envelopes sequentially numbered from 1 to 72 will be prepared according to the random number table. Allocation concealment will be respected.5.6. Criteria and procedures for selecting, capturing and monitoring participants5.6.1. Inclusion criteriaChildren of pre-school age ASA I or II Children undergoing general anesthesia for elective surgery 5.6.2. Exclusion criteria: Pre-existing parenchymal pulmonary disease; Cyanotic children with oxyhemoglobin saturation <95% prior to anesthetic induction; Recent history (<4 weeks) or duration of upper respiratory tract infection; Procedures for Capture and Follow-up of Participants The captation of participants will be carried out by a student of scientific initiation who will not participate in the collection of data. It will be solely responsible for recruiting participants, applying the eligibility criteria using a checklist (Appendix 1) and requesting the signature of the TCLE. This step will happen at the entrance of the surgical block where the patients and accompanying persons, coming from the ambulatory after weighing and measuring, wait for the surgery. Subsequently, it will deliver to the main researcher the envelope referring to the participant where the group to which it is allocated will be included in the interior. The allocation will be made by sequentially numbered, otherwise identical, sealed envelopes, each containing 2 inches by 2 inches with a written code that will designate the intervention group or the comparative group. There will be no detectable differences in size or weight between the envelopes in the intervention group and the envelopes in the comparison group. The envelopes will be opaque and opened sequentially only after writing the information about the patients to whom they are assigned. The envelope opening will occur before the patient enters the operating room so that the study scenario is assembled. The principal investigator will be responsible for the entire procedure, along with the anesthetist responsible for the surgery. Data collection in turn will be performed by a student of scientific initiation responsible only for the collection of data without knowledge of the group to which the patient will be allocated (CPAP system will be selected before the student enters the room for collection). It will be filled a flowchart (CONSORT) with the progress of the study throughout the phases of a parallel intervention study of two groups (participant selection, intervention allocation, monitoring and data analysis). All records of the participants involved in the study will be identified by self-addressed labels containing the research name, registration number, patient identification number in the study and the group in which it was allocated.

Performing anesthesia: Patients selected for the study will be admitted to the operating room and will receive routine monitoring (cardioscope, oximeter, noninvasive blood pressure and capnography). Inhaled induction will be with sevoflurane at 8%, inspired fraction of oxygen at 60, 5% under a flow of fresh gases of 4l / min (2l of oxygen and 2l of compressed air) until loss of the eyelid reflex. The anesthetic concentration will then be reduced to 4%. A facial mask attached to the anesthesia device will be used, fixed to the patient through an elastic band. After adequate ventilation is established through the correct positioning of the facial mask and the present capnography curve, peripheral venous access will be obtained with venous catheter number 20, 22 or 24G for hydration and infusion of propofol at the dose of 3.5mg / kg to induce apnea in patients of both groups.5.9.2. Performing the CPAP The patients will be submitted soon after the monitoring to the technique described in the envelopes delivered at the entrance of the surgical block. In the CPAP group, this will be applied in the anesthesia device (Dräger Fabius GS) using a circular system. This system consists of two corrugated tubes coupled at one end to a Y-piece, connected to the patient's face mask, and at the other end to the anesthesia apparatus which, in addition to providing fresh gas flow, has a carbonic gas absorber called lime soda The latter, besides allowing the system to be circulated by withdrawing CO2 from the air supplied to the patient, heats and humidifies the gas mixture. A pressure relief valve (pop-off valve), which prevents the loss of gases by the patient-device system when closed, is an integral part of the anesthesia machine. It has several markings (0-70cmH2O), is manipulated manually and may be open (0cmH2O), ie without any pressure being supplied to the patient's airway, or closed. In the closed position, there will be a continuous positive pressure being supplied to the patient's airway. The pressure that will be used in this group will be 10cmH2O. In the Open System group, the system will remain with the valve in the open position, that is, 0cmH2O. In both groups, patients will spontaneously ventilate from the beginning with the technique defined at the time of All anesthetic procedure will have the presence of the anesthesiologist who accompanies the patient and will be at his discretion as soon as the study is finished.5.10. Procedures for data collection5.10.1. Data collection instrument Data will be collected using a standardized, pre-coded form for data entry into the computer (Appendix 3). The categorical variables information will be pre-coded and the continuous variables expressed in their own numerical value and only at the time of analysis will the results of some of these be categorized. These forms will be properly stored in specific file folders, before and after typing and analysis, under the responsibility of the researcher himself, who will complete them at different times, before, during and after the surgical procedure.5.10.2. Data collection Data will be collected by an independent researcher who will be present in the operating room, complete the form with patient identification data and study variables and will not interfere with the anesthetic procedure that should be performed. The time from the moment of the cessation of s respiratory and decay curve of capnography will be timed. The measurement will take place until the recording in the pulse oximetry of the value of 95%. Assisted ventilation will then be established (in the Open System group, the pop-off valve will be manually closed up to the value of 10cmH2O). At this time there will be a new timing of the time until reading in the pulse oximetry of the value of 100% or of the value obtained just before the beginning of the apnea.5.11. Processing and analysis of data5.11.1. Data processing The typing in the specific database created in the excel program will be performed twice, in times and by different people, obtaining at the end a listing for correction of possible typos, with supervision of the researcher himself. inconsistencies or lack of data when reviewing the listings, the corresponding archived forms will be consulted according to the patient registration number. At the end of the entry of all the forms in the database, the final review will be done and the missing data will be completed by the processes mentioned above. The definitive database thus created will then be used for statistical analysis in the STATA program, and will also be subjected to consistency tests and information cleaning, generating backup copies.5.11.2. Data analysis Data analysis will be performed by the researcher and the statistician responsible (supervised by her supervisor) using the STATA program. Descriptive statistical analysis will be carried out through measures of central tendency and dispersion for the quantitative variables and through distribution of frequencies for the qualitative variables. For comparison of paired samples, a non-parametric method, Wilcoxcon's T test will be used. Student's t-test will be used for two independent samples, previously considering the Kolgomorov-Smirnoff normality test for each sample, and in the case of non-normality the non-parametric Mann-Whitney U test will be used. The quantitative variables according to the time of their implementation will be used analysis of variance for the repeated units (ANOVA), if they present a normal distribution, or Wilcoxcon if the normality is not present. For categorical variables Fisher's exact test or chi-square test will be used, when necessary. The Relative Risk and its 95% confidence interval, in addition to the Number Needed to Treat and obtain a benefit (NNT) or Number Required to Treat or obtain a harm (NNH) when relevant, are also calculated.5.12. Ethical AspectsThe research will respect human rights and the principles of bioethics (Autonomy, Non-Maleficence, Charity, Justice and Equity). The confidentiality and confidentiality in the collection and archiving of the data collected will be respected. The terms of the National Health Council Resolution No. 466 of December 12, 2012 for research on human beings will be followed as well as the Declaration of Helsinki. In addition, the project will be submitted to the Research Ethics Committee of the proposing institution, data will only be collected after such submission and approval of said committee and the children of the study will only be included after the responsible sign the consent form and The study will be registered at ClinicalTrials.gov and will not offer additional risks or discomforts, in addition to those inherent to the anesthetic-surgical procedure itself, nor have there been any reported adverse effects in the literature that contraindicate the use of CPAP during induction. On the contrary, the few existing studies report respiratory improvement with the use of this intervention. All research procedures will be carried out by trained and qualified professionals, both for pediatric anesthesia and CPAP. The TCLE (Appendix 2) will share with the caregivers all the information regarding the advantages and disadvantages of using the two techniques; emphasize that no routine procedure will be performed by the team; and it will include the right to refuse to participate in the study, as well as the guarantee of assistance to those who do not accept, without reimbursement by the institution, nor by the researchers. They will undertake to publish the study irrespective of the results obtained.5.13. Conflicts of interest This research will be free of conflict of interest, private or institutional. There will be no funding from the pharmaceutical industry or representatives of any research object used.
