# Supplementary material for: Continuous positive airway pressure to reduce the risk of early peripheral oxygen desaturation after onset of apnoea in children: A double-blind randomised controlled trial
Source: PLoS One. 2021 Oct 1;16(10):e0256950. doi: 10.1371/journal.pone.0256950 (PMC8486132; doi:10.1371/journal.pone.0256950)
Supplement: S4 File — Database containing each CPAP group patients time to a SpO2 of 95% or 300 seconds. (PDF) [file pone.0256950.s007.pdf]

Paciente	Grupo	Tempo	Status	
1	1	300	0	
2	1	201	1	
4	1	142	1	
7	1	300	0	
9	1	300	0	
10	1	300	0	
12	1	277	1	
15	1	300	0	
17	1	210	1	
18	1	230	1	
20	1	140	1	
26	1	300	0	
28	1	300	0	
31	1	120	1	
33	1	300	0	
34	1	300	0	
36	1	300	0	
39	1	300	0	
41	1	300	0	
42	1	75	1	
44	1	300	0	
47	1	225	1	
49	1	90	1	
50	1	300	0	
52	1	202	1	
55	1	300	0	
57	1	300	0	
58	1	80	1	
60	1	130	1	
63	1	147	1	
65	1	300	0	
66	1	177	1	
68	1	112	1	
71	1	82	1	
