# Supplementary material for: Continuous positive airway pressure to reduce the risk of early peripheral oxygen desaturation after onset of apnoea in children: A double-blind randomised controlled trial
Source: PLoS One. 2021 Oct 1;16(10):e0256950. doi: 10.1371/journal.pone.0256950 (PMC8486132; doi:10.1371/journal.pone.0256950)
Supplement: S5 File — Database containing each Control group patients time to a SpO2 of 95% or 300 seconds. (PDF) [file pone.0256950.s008.pdf]

Paciente	Grupo	Tempo	Status	
3	2	44	1	
5	2	100	1	
8	2	125	1	
11	2	100	1	
13	2	132	1	
14	2	58	1	
16	2	230	1	
19	2	76	1	
21	2	300	0	
22	2	110	1	
24	2	140	1	
27	2	157	1	
29	2	87	1	
30	2	248	1	
32	2	60	1	
35	2	100	1	
37	2	30	1	
38	2	220	1	
40	2	200	1	
43	2	190	1	
45	2	80	1	
46	2	140	1	
48	2	300	0	
51	2	150	1	
53	2	90	1	
54	2	140	1	
56	2	200	1	
59	2	30	1	
61	2	78	1	
62	2	90	1	
64	2	85	1	
67	2	118	1	
70	2	183	1	
72	2	154	1	
