# Supplementary material for: Continuous positive airway pressure to reduce the risk of early peripheral oxygen desaturation after onset of apnoea in children: A double-blind randomised controlled trial
Source: PLoS One. 2021 Oct 1;16(10):e0256950. doi: 10.1371/journal.pone.0256950 (PMC8486132; doi:10.1371/journal.pone.0256950)
Supplement: S6 File — Database containing each patient SpO2 during apnoea time until SpO2 of 95% or 300 seconds. (PDF) [file pone.0256950.s009.pdf]

Paciente	Tempo	Sat	Grupo	
1	0	100	1	
1	10	100	1	
1	20	100	1	
1	30	100	1	
1	40	100	1	
1	50	100	1	
1	60	100	1	
1	70	100	1	
1	80	100	1	
1	90	100	1	
1	100	100	1	
1	110	100	1	
1	120	100	1	
1	130	100	1	
1	140	100	1	
1	150	100	1	
1	160	100	1	
1	170	100	1	
1	180	100	1	
1	190	100	1	
1	200	100	1	
1	210	100	1	
1	220	100	1	
1	230	100	1	
1	240	100	1	
1	250	100	1	
1	260	100	1	
1	270	100	1	
1	280	100	1	
1	290	100	1	
1	300	100	1	
2	0	100	1	
2	10	100	1	
2	20	100	1	
2	30	100	1	
2	40	100	1	
2	50	100	1	
2	60	100	1	
2	70	100	1	
2	80	100	1	
2	90	100	1	
2	100	100	1	
2	110	100	1	
2	120	100	1	
2	130	100	1	
2	140	100	1	
2	150	100	1	
2	160	99	1	
2	170	99	1	
2	180	98	1	
2	190	97	1	

2	200	96	1	
2	201	95	1	
4	0	100	1	
4	10	100	1	
4	20	99	1	
4	30	99	1	
4	40	99	1	
4	50	99	1	
4	60	99	1	
4	70	99	1	
4	80	99	1	
4	90	99	1	
4	100	99	1	
4	110	98	1	
4	120	97	1	
4	130	96	1	
4	140	96	1	
4	142	95	1	
7	0	99	1	
7	10	99	1	
7	20	99	1	
7	30	98	1	
7	40	98	1	
7	50	98	1	
7	60	98	1	
7	70	98	1	
7	80	98	1	
7	90	98	1	
7	100	97	1	
7	110	98	1	
7	120	97	1	
7	130	97	1	
7	140	98	1	
7	150	97	1	
7	160	97	1	
7	170	97	1	
7	180	97	1	
7	190	97	1	
7	200	97	1	
7	210	97	1	
7	220	97	1	
7	230	97	1	
7	240	97	1	
7	250	97	1	
7	260	97	1	
7	270	97	1	
7	280	97	1	
7	290	97	1	
7	300	97	1	
9	0	100	1	
9	10	100	1	
9	20	99	1	

9	30	99	1	
9	40	99	1	
9	50	99	1	
9	60	99	1	
9	70	98	1	
9	80	98	1	
9	90	98	1	
9	100	98	1	
9	110	98	1	
9	120	98	1	
9	130	98	1	
9	140	98	1	
9	150	98	1	
9	160	98	1	
9	170	98	1	
9	180	98	1	
9	190	98	1	
9	200	98	1	
9	210	98	1	
9	220	98	1	
9	230	98	1	
9	240	98	1	
9	250	98	1	
9	260	98	1	
9	270	98	1	
9	280	98	1	
9	290	98	1	
9	300	98	1	
10	0	99	1	
10	10	99	1	
10	20	99	1	
10	30	99	1	
10	40	99	1	
10	50	99	1	
10	60	99	1	
10	70	99	1	
10	80	99	1	
10	90	99	1	
10	100	99	1	
10	110	99	1	
10	120	99	1	
10	130	99	1	
10	140	99	1	
10	150	99	1	
10	160	99	1	
10	170	99	1	
10	180	99	1	
10	190	99	1	
10	200	99	1	
10	210	99	1	
10	220	99	1	
10	230	99	1	

10	240	99	1	
10	250	99	1	
10	260	99	1	
10	270	99	1	
10	280	99	1	
10	290	99	1	
10	300	99	1	
12	0	100	1	
12	10	99	1	
12	20	99	1	
12	30	99	1	
12	40	99	1	
12	50	99	1	
12	60	99	1	
12	70	99	1	
12	80	99	1	
12	90	99	1	
12	100	99	1	
12	110	99	1	
12	120	98	1	
12	130	98	1	
12	140	98	1	
12	150	98	1	
12	160	98	1	
12	170	98	1	
12	180	98	1	
12	190	98	1	
12	200	97	1	
12	210	97	1	
12	220	97	1	
12	230	96	1	
12	240	96	1	
12	250	96	1	
12	260	96	1	
12	270	96	1	
12	277	95	1	
15	0	99	1	
15	10	99	1	
15	20	99	1	
15	30	99	1	
15	40	99	1	
15	50	99	1	
15	60	99	1	
15	70	99	1	
15	80	99	1	
15	90	99	1	
15	100	99	1	
15	110	99	1	
15	120	99	1	
15	130	99	1	
15	140	99	1	
15	150	99	1	

15	160	99	1	
15	170	99	1	
15	180	99	1	
15	190	99	1	
15	200	99	1	
15	210	99	1	
15	220	99	1	
15	230	99	1	
15	240	99	1	
15	250	99	1	
15	260	99	1	
15	270	99	1	
15	280	99	1	
15	290	99	1	
15	300	99	1	
17	0	99	1	
17	10	99	1	
17	20	99	1	
17	30	99	1	
17	40	99	1	
17	50	99	1	
17	60	99	1	
17	70	99	1	
17	80	99	1	
17	90	98	1	
17	100	98	1	
17	110	98	1	
17	120	98	1	
17	130	98	1	
17	140	98	1	
17	150	98	1	
17	160	97	1	
17	170	97	1	
17	180	97	1	
17	190	96	1	
17	200	96	1	
17	210	95	1	
18	0	100	1	
18	10	100	1	
18	20	100	1	
18	30	100	1	
18	40	100	1	
18	50	100	1	
18	60	100	1	
18	70	100	1	
18	80	99	1	
18	90	99	1	
18	100	99	1	
18	110	99	1	
18	120	99	1	
18	130	99	1	
18	140	99	1	

18	150	99	1	
18	160	99	1	
18	170	98	1	
18	180	98	1	
18	190	98	1	
18	200	98	1	
18	210	97	1	
18	220	96	1	
18	230	95	1	
20	0	99	1	
20	10	98	1	
20	20	98	1	
20	30	98	1	
20	40	98	1	
20	50	98	1	
20	60	98	1	
20	70	98	1	
20	80	98	1	
20	90	98	1	
20	100	97	1	
20	110	97	1	
20	120	97	1	
20	130	96	1	
20	140	95	1	
26	0	100	1	
26	10	100	1	
26	20	100	1	
26	30	100	1	
26	40	100	1	
26	50	100	1	
26	60	99	1	
26	70	100	1	
26	80	100	1	
26	90	100	1	
26	100	100	1	
26	110	100	1	
26	120	100	1	
26	130	100	1	
26	140	99	1	
26	150	99	1	
26	160	99	1	
26	170	99	1	
26	180	99	1	
26	190	99	1	
26	200	99	1	
26	210	99	1	
26	220	99	1	
26	230	99	1	
26	240	99	1	
26	250	99	1	
26	260	99	1	
26	270	99	1	

26	280	99	1	
26	290	99	1	
26	300	99	1	
28	0	99	1	
28	10	99	1	
28	20	99	1	
28	30	99	1	
28	40	99	1	
28	50	99	1	
28	60	99	1	
28	70	99	1	
28	80	99	1	
28	90	99	1	
28	100	99	1	
28	110	99	1	
28	120	99	1	
28	130	99	1	
28	140	99	1	
28	150	99	1	
28	160	99	1	
28	170	99	1	
28	180	99	1	
28	190	99	1	
28	200	99	1	
28	210	99	1	
28	220	99	1	
28	230	99	1	
28	240	99	1	
28	250	99	1	
28	260	99	1	
28	270	99	1	
28	280	99	1	
28	290	99	1	
28	300	99	1	
31	0	99	1	
31	10	99	1	
31	20	98	1	
31	30	98	1	
31	40	98	1	
31	50	98	1	
31	60	98	1	
31	70	97	1	
31	80	97	1	
31	90	96	1	
31	100	96	1	
31	110	96	1	
31	120	95	1	
33	0	99	1	
33	10	99	1	
33	20	99	1	
33	30	99	1	
33	40	99	1	

33	50	99	1	
33	60	99	1	
33	70	99	1	
33	80	99	1	
33	90	99	1	
33	100	99	1	
33	110	99	1	
33	120	99	1	
33	130	99	1	
33	140	99	1	
33	150	99	1	
33	160	99	1	
33	170	99	1	
33	180	99	1	
33	190	99	1	
33	200	99	1	
33	210	99	1	
33	220	99	1	
33	230	99	1	
33	240	98	1	
33	250	98	1	
33	260	98	1	
33	270	98	1	
33	280	98	1	
33	290	98	1	
33	300	98	1	
34	0	99	1	
34	10	99	1	
34	20	99	1	
34	30	99	1	
34	40	99	1	
34	50	99	1	
34	60	99	1	
34	70	99	1	
34	80	99	1	
34	90	99	1	
34	100	99	1	
34	110	99	1	
34	120	99	1	
34	130	99	1	
34	140	99	1	
34	150	99	1	
34	160	98	1	
34	170	98	1	
34	180	98	1	
34	190	98	1	
34	200	98	1	
34	210	98	1	
34	220	98	1	
34	230	98	1	
34	240	98	1	
34	250	98	1	

34	260	98	1	
34	270	98	1	
34	280	98	1	
34	290	98	1	
34	300	98	1	
36	0	99	1	
36	10	99	1	
36	20	99	1	
36	30	99	1	
36	40	99	1	
36	50	99	1	
36	60	99	1	
36	70	99	1	
36	80	99	1	
36	90	99	1	
36	100	99	1	
36	110	99	1	
36	120	99	1	
36	130	99	1	
36	140	99	1	
36	150	99	1	
36	160	99	1	
36	170	99	1	
36	180	99	1	
36	190	99	1	
36	200	99	1	
36	210	99	1	
36	220	99	1	
36	230	99	1	
36	240	99	1	
36	250	99	1	
36	260	99	1	
36	270	99	1	
36	280	98	1	
36	290	98	1	
36	300	98	1	
39	0	100	1	
39	10	100	1	
39	20	100	1	
39	30	100	1	
39	40	100	1	
39	50	99	1	
39	60	99	1	
39	70	99	1	
39	80	99	1	
39	90	99	1	
39	100	99	1	
39	110	99	1	
39	120	99	1	
39	130	99	1	
39	140	99	1	
39	150	99	1	

39	160	99	1	
39	170	99	1	
39	180	99	1	
39	190	99	1	
39	200	99	1	
39	210	99	1	
39	220	99	1	
39	230	99	1	
39	240	99	1	
39	250	99	1	
39	260	99	1	
39	270	99	1	
39	280	99	1	
39	290	99	1	
39	300	99	1	
41	0	100	1	
41	10	100	1	
41	20	100	1	
41	30	100	1	
41	40	100	1	
41	50	100	1	
41	60	100	1	
41	70	100	1	
41	80	100	1	
41	90	100	1	
41	100	100	1	
41	110	100	1	
41	120	100	1	
41	130	100	1	
41	140	100	1	
41	150	100	1	
41	160	100	1	
41	170	100	1	
41	180	100	1	
41	190	100	1	
41	200	100	1	
41	210	100	1	
41	220	100	1	
41	230	100	1	
41	240	100	1	
41	250	100	1	
41	260	100	1	
41	270	100	1	
41	280	100	1	
41	290	100	1	
41	300	100	1	
42	0	100	1	
42	10	99	1	
42	20	99	1	
42	30	99	1	
42	40	98	1	
42	50	97	1	

42	60	97	1	
42	70	96	1	
42	75	95	1	
44	0	100	1	
44	10	100	1	
44	20	98	1	
44	30	99	1	
44	40	99	1	
44	50	99	1	
44	60	98	1	
44	70	98	1	
44	80	98	1	
44	90	98	1	
44	100	98	1	
44	110	99	1	
44	120	99	1	
44	130	99	1	
44	140	99	1	
44	150	99	1	
44	160	99	1	
44	170	99	1	
44	180	99	1	
44	190	99	1	
44	200	99	1	
44	210	99	1	
44	220	99	1	
44	230	99	1	
44	240	99	1	
44	250	99	1	
44	260	99	1	
44	270	99	1	
44	280	99	1	
44	290	99	1	
44	300	99	1	
47	0	100	1	
47	10	100	1	
47	20	100	1	
47	30	100	1	
47	40	100	1	
47	50	100	1	
47	60	100	1	
47	70	100	1	
47	80	100	1	
47	90	100	1	
47	100	100	1	
47	110	100	1	
47	120	100	1	
47	130	100	1	
47	140	100	1	
47	150	99	1	
47	160	99	1	
47	170	99	1	

47	180	99	1	
47	190	98	1	
47	200	97	1	
47	210	96	1	
47	220	96	1	
47	225	95	1	
49	0	99	1	
49	10	100	1	
49	20	100	1	
49	30	100	1	
49	40	99	1	
49	50	99	1	
49	60	98	1	
49	70	97	1	
49	80	96	1	
49	90	95	1	
50	0	100	1	
50	10	100	1	
50	20	100	1	
50	30	100	1	
50	40	100	1	
50	50	100	1	
50	60	100	1	
50	70	100	1	
50	80	100	1	
50	90	100	1	
50	100	100	1	
50	110	100	1	
50	120	100	1	
50	130	100	1	
50	140	100	1	
50	150	100	1	
50	160	100	1	
50	170	100	1	
50	180	100	1	
50	190	100	1	
50	200	100	1	
50	210	100	1	
50	220	100	1	
50	230	100	1	
50	240	100	1	
50	250	100	1	
50	260	100	1	
50	270	100	1	
50	280	100	1	
50	290	100	1	
50	300	100	1	
52	0	99	1	
52	10	99	1	
52	20	99	1	
52	30	99	1	
52	40	99	1	

52	50	99	1	
52	60	99	1	
52	70	99	1	
52	80	99	1	
52	90	99	1	
52	100	99	1	
52	110	99	1	
52	120	99	1	
52	130	98	1	
52	140	98	1	
52	150	98	1	
52	160	98	1	
52	170	98	1	
52	180	97	1	
52	190	96	1	
52	200	96	1	
52	202	95	1	
55	0	99	1	
55	10	99	1	
55	20	99	1	
55	30	99	1	
55	40	99	1	
55	50	99	1	
55	60	99	1	
55	70	99	1	
55	80	99	1	
55	90	99	1	
55	100	99	1	
55	110	99	1	
55	120	99	1	
55	130	99	1	
55	140	99	1	
55	150	98	1	
55	160	98	1	
55	170	98	1	
55	180	98	1	
55	190	98	1	
55	200	98	1	
55	210	98	1	
55	220	98	1	
55	230	98	1	
55	240	98	1	
55	250	98	1	
55	260	98	1	
55	270	98	1	
55	280	98	1	
55	290	98	1	
55	300	98	1	
57	0	99	1	
57	10	100	1	
57	20	100	1	
57	30	100	1	

57	40	100	1	
57	50	100	1	
57	60	100	1	
57	70	100	1	
57	80	100	1	
57	90	100	1	
57	100	100	1	
57	110	100	1	
57	120	99	1	
57	130	98	1	
57	140	98	1	
57	150	99	1	
57	160	99	1	
57	170	99	1	
57	180	99	1	
57	190	99	1	
57	200	99	1	
57	210	99	1	
57	220	99	1	
57	230	99	1	
57	240	99	1	
57	250	99	1	
57	260	99	1	
57	270	99	1	
57	280	99	1	
57	290	99	1	
57	300	99	1	
58	0	100	1	
58	10	99	1	
58	20	99	1	
58	30	99	1	
58	40	99	1	
58	50	99	1	
58	60	98	1	
58	70	97	1	
58	80	95	1	
60	0	99	1	
60	10	99	1	
60	20	98	1	
60	30	98	1	
60	40	99	1	
60	50	98	1	
60	60	98	1	
60	70	98	1	
60	80	97	1	
60	90	97	1	
60	100	96	1	
60	110	97	1	
60	120	97	1	
60	130	95	1	
63	0	99	1	
63	10	99	1	

63	20	99	1	
63	30	99	1	
63	40	99	1	
63	50	99	1	
63	60	99	1	
63	70	99	1	
63	80	99	1	
63	90	99	1	
63	100	99	1	
63	110	99	1	
63	120	99	1	
63	130	98	1	
63	140	97	1	
63	147	95	1	
65	0	99	1	
65	10	99	1	
65	20	99	1	
65	30	99	1	
65	40	99	1	
65	50	99	1	
65	60	99	1	
65	70	99	1	
65	80	99	1	
65	90	99	1	
65	100	99	1	
65	110	99	1	
65	120	99	1	
65	130	99	1	
65	140	99	1	
65	150	99	1	
65	160	99	1	
65	170	99	1	
65	180	99	1	
65	190	99	1	
65	200	99	1	
65	210	99	1	
65	220	99	1	
65	230	99	1	
65	240	99	1	
65	250	99	1	
65	260	99	1	
65	270	99	1	
65	280	99	1	
65	290	99	1	
65	300	99	1	
66	0	100	1	
66	10	100	1	
66	20	99	1	
66	30	99	1	
66	40	99	1	
66	50	99	1	
66	60	99	1	

66	70	99	1	
66	80	99	1	
66	90	99	1	
66	100	99	1	
66	110	98	1	
66	120	98	1	
66	130	98	1	
66	140	98	1	
66	150	98	1	
66	160	97	1	
66	170	96	1	
66	177	95	1	
68	0	99	1	
68	10	99	1	
68	20	99	1	
68	30	99	1	
68	40	99	1	
68	50	99	1	
68	60	99	1	
68	70	99	1	
68	80	99	1	
68	90	98	1	
68	100	97	1	
68	110	96	1	
68	112	95	1	
71	0	98	1	
71	10	98	1	
71	20	98	1	
71	30	98	1	
71	40	97	1	
71	50	97	1	
71	60	97	1	
71	70	97	1	
71	80	96	1	
71	82	95	1	
3	0	98	2	
3	10	98	2	
3	20	98	2	
3	30	97	2	
3	40	96	2	
3	44	95	2	
5	0	100	2	
5	10	100	2	
5	20	99	2	
5	30	99	2	
5	40	99	2	
5	50	98	2	
5	60	98	2	
5	70	98	2	
5	80	97	2	
5	90	96	2	
5	100	95	2	

8	0	100	2	
8	10	100	2	
8	20	100	2	
8	30	100	2	
8	40	100	2	
8	50	100	2	
8	60	100	2	
8	70	100	2	
8	80	100	2	
8	90	100	2	
8	100	99	2	
8	110	98	2	
8	120	96	2	
8	125	95	2	
11	0	100	2	
11	10	100	2	
11	20	100	2	
11	30	100	2	
11	40	100	2	
11	50	99	2	
11	60	99	2	
11	70	98	2	
11	80	98	2	
11	90	97	2	
11	100	95	2	
13	0	99	2	
13	10	99	2	
13	20	99	2	
13	30	98	2	
13	40	98	2	
13	50	98	2	
13	60	98	2	
13	70	98	2	
13	80	98	2	
13	90	97	2	
13	100	97	2	
13	110	96	2	
13	120	96	2	
13	130	96	2	
13	132	95	2	
14	0	100	2	
14	10	100	2	
14	20	100	2	
14	30	98	2	
14	40	97	2	
14	50	96	2	
14	58	95	2	
16	0	100	2	
16	10	100	2	
16	20	100	2	
16	30	100	2	
16	40	100	2	

16	50	100	2	
16	60	100	2	
16	70	100	2	
16	80	100	2	
16	90	100	2	
16	100	100	2	
16	110	99	2	
16	120	99	2	
16	130	99	2	
16	140	99	2	
16	150	99	2	
16	160	98	2	
16	170	98	2	
16	180	98	2	
16	190	98	2	
16	200	97	2	
16	210	96	2	
16	220	96	2	
16	230	95	2	
19	0	97	2	
19	10	97	2	
19	20	97	2	
19	30	97	2	
19	40	97	2	
19	50	96	2	
19	60	96	2	
19	70	96	2	
19	76	95	2	
21	0	100	2	
21	10	99	2	
21	20	99	2	
21	30	99	2	
21	40	99	2	
21	50	99	2	
21	60	99	2	
21	70	99	2	
21	80	99	2	
21	90	99	2	
21	100	99	2	
21	110	99	2	
21	120	99	2	
21	130	99	2	
21	140	99	2	
21	150	99	2	
21	160	99	2	
21	170	99	2	
21	180	99	2	
21	190	99	2	
21	200	99	2	
21	210	99	2	
21	220	99	2	
21	230	99	2	

21	240	99	2	
21	250	99	2	
21	260	99	2	
21	270	99	2	
21	280	99	2	
21	290	99	2	
21	300	99	2	
22	0	100	2	
22	10	100	2	
22	20	100	2	
22	30	100	2	
22	40	100	2	
22	50	100	2	
22	60	100	2	
22	70	99	2	
22	80	98	2	
22	90	97	2	
22	100	96	2	
22	110	95	2	
24	0	100	2	
24	10	100	2	
24	20	100	2	
24	30	100	2	
24	40	100	2	
24	50	100	2	
24	60	99	2	
24	70	99	2	
24	80	99	2	
24	90	99	2	
24	100	99	2	
24	110	98	2	
24	120	97	2	
24	130	96	2	
24	140	95	2	
27	0	100	2	
27	10	100	2	
27	20	99	2	
27	30	99	2	
27	40	99	2	
27	50	99	2	
27	60	99	2	
27	70	99	2	
27	80	99	2	
27	90	99	2	
27	100	99	2	
27	110	98	2	
27	120	98	2	
27	130	97	2	
27	140	97	2	
27	150	96	2	
27	157	95	2	
29	0	100	2	

29	10	99	2	
29	20	99	2	
29	30	99	2	
29	40	99	2	
29	50	98	2	
29	60	98	2	
29	70	97	2	
29	80	96	2	
29	87	95	2	
30	0	99	2	
30	10	98	2	
30	20	98	2	
30	30	98	2	
30	40	98	2	
30	50	98	2	
30	60	98	2	
30	70	98	2	
30	80	97	2	
30	90	97	2	
30	100	97	2	
30	110	97	2	
30	120	97	2	
30	130	97	2	
30	140	98	2	
30	150	97	2	
30	160	98	2	
30	170	97	2	
30	180	97	2	
30	190	97	2	
30	200	97	2	
30	210	97	2	
30	220	96	2	
30	230	96	2	
30	240	96	2	
30	248	95	2	
32	0	98	2	
32	10	98	2	
32	20	97	2	
32	30	97	2	
32	40	96	2	
32	50	96	2	
32	60	95	2	
35	0	99	2	
35	10	99	2	
35	20	99	2	
35	30	99	2	
35	40	99	2	
35	50	98	2	
35	60	98	2	
35	70	97	2	
35	80	97	2	
35	90	96	2	

35	100	95	2	
37	0	99	2	
37	10	96	2	
37	20	96	2	
37	30	95	2	
38	0	100	2	
38	10	100	2	
38	20	100	2	
38	30	100	2	
38	40	100	2	
38	50	99	2	
38	60	99	2	
38	70	99	2	
38	80	99	2	
38	90	99	2	
38	100	99	2	
38	110	99	2	
38	120	99	2	
38	130	99	2	
38	140	99	2	
38	150	99	2	
38	160	99	2	
38	170	99	2	
38	180	97	2	
38	190	97	2	
38	200	97	2	
38	210	96	2	
38	220	95	2	
40	0	100	2	
40	10	100	2	
40	20	100	2	
40	30	100	2	
40	40	100	2	
40	50	100	2	
40	60	100	2	
40	70	100	2	
40	80	100	2	
40	90	100	2	
40	100	99	2	
40	110		2	
40	120		2	
40	130		2	
40	140		2	
40	150		2	
40	160		2	
40	170		2	
40	180		2	
40	190		2	
40	200	95	2	
43	0	98	2	
43	10	98	2	
43	20	98	2	

43	30	98	2	
43	40	98	2	
43	50	98	2	
43	60	97	2	
43	70	97	2	
43	80	97	2	
43	90	97	2	
43	100	97	2	
43	110	97	2	
43	120	97	2	
43	130	97	2	
43	140	97	2	
43	150	97	2	
43	160	97	2	
43	170	97	2	
43	180	96	2	
43	190	95	2	
45	0	100	2	
45	10	100	2	
45	20	99	2	
45	30	99	2	
45	40	99	2	
45	50	98	2	
45	60	97	2	
45	70	96	2	
45	80	95	2	
46	0	100	2	
46	10	100	2	
46	20	100	2	
46	30	100	2	
46	40	100	2	
46	50	100	2	
46	60	100	2	
46	70	100	2	
46	80	100	2	
46	90	99	2	
46	100	99	2	
46	110	98	2	
46	120	98	2	
46	130	97	2	
46	140	95	2	
48	0	99	2	
48	10	99	2	
48	20	99	2	
48	30	99	2	
48	40	99	2	
48	50	99	2	
48	60	99	2	
48	70	99	2	
48	80	99	2	
48	90	99	2	
48	100	99	2	

48	110	99	2	
48	120	99	2	
48	130	99	2	
48	140	98	2	
48	150	98	2	
48	160	98	2	
48	170	98	2	
48	180	98	2	
48	190	98	2	
48	200	98	2	
48	210	98	2	
48	220	98	2	
48	230	99	2	
48	240	99	2	
48	250	99	2	
48	260	99	2	
48	270	99	2	
48	280	99	2	
48	290	99	2	
48	300	99	2	
51	0	99	2	
51	10	99	2	
51	20	99	2	
51	30	99	2	
51	40	99	2	
51	50	98	2	
51	60	98	2	
51	70	97	2	
51	80	96	2	
51	90	96	2	
51	100	96	2	
51	110	96	2	
51	120	96	2	
51	130	96	2	
51	140	96	2	
51	150	95	2	
53	0	99	2	
53	10	100	2	
53	20	99	2	
53	30	99	2	
53	40	99	2	
53	50	99	2	
53	60	98	2	
53	70	97	2	
53	80	96	2	
53	90	95	2	
54	0	100	2	
54	10	100	2	
54	20	100	2	
54	30	100	2	
54	40	100	2	
54	50	100	2	

54	60	100	2	
54	70	100	2	
54	80	100	2	
54	90	100	2	
54	100	99	2	
54	110	99	2	
54	120	98	2	
54	130	96	2	
54	140	95	2	
56	0	100	2	
56	10	100	2	
56	20	100	2	
56	30	100	2	
56	40	100	2	
56	50	100	2	
56	60	100	2	
56	70	100	2	
56	80	100	2	
56	90	100	2	
56	100	100	2	
56	110	100	2	
56	120	100	2	
56	130	100	2	
56	140	100	2	
56	150	99	2	
56	160	98	2	
56	170	98	2	
56	180	97	2	
56	190	96	2	
56	200	95	2	
59	0	99	2	
59	10	98	2	
59	20	97	2	
59	30	95	2	
61	0	100	2	
61	10	100	2	
61	20	99	2	
61	30	99	2	
61	40	98	2	
61	50	98	2	
61	60	97	2	
61	70	96	2	
61	78	95	2	
62	0	99	2	
62	10	99	2	
62	20	99	2	
62	30	99	2	
62	40	99	2	
62	50	98	2	
62	60	97	2	
62	70	97	2	
62	80	97	2	

62	90	95	2	
64	0	99	2	
64	10	99	2	
64	20	99	2	
64	30	98	2	
64	40	98	2	
64	50	97	2	
64	60	97	2	
64	70	96	2	
64	80	96	2	
64	85	95	2	
67	0	99	2	
67	10	99	2	
67	20	99	2	
67	30	99	2	
67	40	99	2	
67	50	99	2	
67	60	99	2	
67	70	99	2	
67	80	99	2	
67	90	98	2	
67	100	97	2	
67	110	96	2	
67	118	95	2	
70	0	99	2	
70	10	99	2	
70	20	99	2	
70	30	99	2	
70	40	99	2	
70	50	99	2	
70	60	99	2	
70	70	99	2	
70	80	99	2	
70	90	99	2	
70	100	99	2	
70	110	99	2	
70	120	99	2	
70	130	99	2	
70	140	99	2	
70	150	99	2	
70	160	99	2	
70	170	98	2	
70	180	97	2	
70	183	95	2	
72	0	99	2	
72	10	99	2	
72	20	99	2	
72	30	99	2	
72	40	99	2	
72	50	99	2	
72	60	99	2	
72	70	99	2	

72	80	99	2	
72	90	99	2	
72	100	97	2	
72	110	97	2	
72	120	97	2	
72	130	97	2	
72	140	97	2	
72	150	96	2	
72	154	95	2	
