# Supplementary material for: Continuous positive airway pressure to reduce the risk of early peripheral oxygen desaturation after onset of apnoea in children: A double-blind randomised controlled trial
Source: PLoS One. 2021 Oct 1;16(10):e0256950. doi: 10.1371/journal.pone.0256950 (PMC8486132; doi:10.1371/journal.pone.0256950)
Supplement: S7 File — Database containing each CPAP group patients time required for recovery of SpO2 from 95% to pre-apnoea levels (T2). (PDF) [file pone.0256950.s010.pdf]

Paciente	Grupo	Tempo2
1	1	0
2	1	20
4	1	21
7	1	0
9	1	0
10	1	0
12	1	21
15	1	0
17	1	32
18	1	43
20	1	170
26	1	0
28	1	0
31	1	30
33	1	0
34	1	0
36	1	0
39	1	0
41	1	
42	1	10
44	1	0
47	1	25
49	1	30
50	1	0
52	1	10
55	1	0
57	1	0
58	1	20
60	1	17
63	1	13
65	1	0
66	1	15
68	1	16
71	1	164
