# Supplementary material for: Continuous positive airway pressure to reduce the risk of early peripheral oxygen desaturation after onset of apnoea in children: A double-blind randomised controlled trial
Source: PLoS One. 2021 Oct 1;16(10):e0256950. doi: 10.1371/journal.pone.0256950 (PMC8486132; doi:10.1371/journal.pone.0256950)
Supplement: S8 File — Database containing each Control group patients time required for recovery of SpO2 from 95% to pre-apnoea levels (T2). (PDF) [file pone.0256950.s011.pdf]

Paciente	Grupo	Tempo2
3	2	24
5	2	25
8	2	19
11	2	22
13	2	45
14	2	20
16	2	15
19	2	17
21	2	0
22	2	26
24	2	29
27	2	88
29	2	77
30	2	48
32	2	33
35	2	14
37	2	10
38	2	40
40	2	60
43	2	55
45	2	45
46	2	30
48	2	0
51	2	18
53	2	22
54	2	13
56	2	27
59	2	15
61	2	35
62	2	38
64	2	50
67	2	31
70	2	29
72	2	360
