# Supplementary material for: Continuous positive airway pressure to reduce the risk of early peripheral oxygen desaturation after onset of apnoea in children: A double-blind randomised controlled trial
Source: PLoS One. 2021 Oct 1;16(10):e0256950. doi: 10.1371/journal.pone.0256950 (PMC8486132; doi:10.1371/journal.pone.0256950)
Supplement: S9 File — (PDF) [file pone.0256950.s012.pdf]

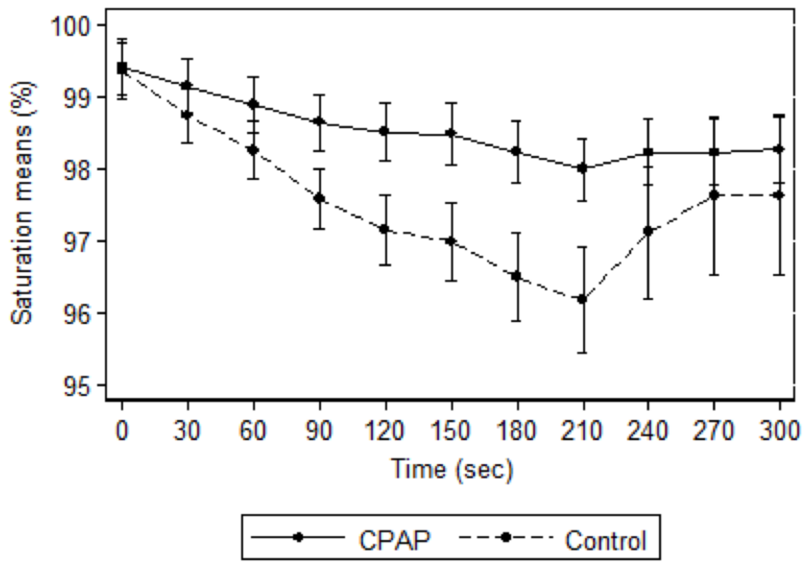

Time	CPAP Mean ± SD	Control Mean ± SD	Mean difference* (CI95%)	P	P adjus-ted†
0	99.4 ± 0.20	99.4 ± 0.20	0.1(-0.49 to 0.60)	0.832	0.832
30	99.1 ± 0.20	98.7 ± 0.20	0.4(-0.13 a 0.96)	0.138	0.190
60	98.9 ± 0.20	98.3 ± 0.20	0.6(0.08 a 1.18)	0.026	0.047
90	98.6 ± 0.20	97.6 ± 0.22	1.1(0.48 a 1.64)	<0.001	0.001
120	98.5 ± 0.20	97.1 ± 0.24	1.4(0.74 a 1.98)	<0.001	< 0.001
150	98.5 ± 0.21	97.0 ± 0.28	1.5(0.81 a 2.17)	<0.001	< 0.001
180	98.2 ± 0.22	96.5 ± 0.31	1.7(0.99 a 2.48)	<0.001	< 0.001
210	98.0 ± 0.22	96.2 ± 0.37	1.8(0.96 a 2.66)	<0.001	< 0.001
240	98.2 ± 0.23	97.1 ± 0.47	1.1(0.09 a 2.13)	0.034	0.053
270	98.2 ± 0.23	97.6 ± 0.56	0.6(-0.59 a 1.79)	0.323	0.355
300	98.3 ± 0.24	97.6 ± 0.56	0.6(-0.55 a 1.84)	0.292	0.355

*Mean difference: CPAP – Control; † Benjamini – Hochberg Method.
