# Supplementary material for: Continuous positive airway pressure to reduce the risk of early peripheral oxygen desaturation after onset of apnoea in children: A double-blind randomised controlled trial
Source: PLoS One. 2021 Oct 1;16(10):e0256950. doi: 10.1371/journal.pone.0256950 (PMC8486132; doi:10.1371/journal.pone.0256950)
Supplement: S11 File — (PDF) [file pone.0256950.s014.pdf]

I. INTRODUCTION

General anaesthesia can be defined as a transitory stage of unconsciousness induced by drugs through their actions on molecular receptors.[1,2] Little is known about its mechanisms, but it is assumed that this state is an adaptive phenomenon, similar to sleep.[1] It is a reversible condition that presents specific behavioural and physiological patterns (unconsciousness, amnesia, analgesia and akinesia), and consists of three phases: induction, maintenance, and emergency or awakening.[2]

Anaesthetic induction is the period in which hypnotic drugs (venous, inhaled, or a combination of both)[2,3] are administered. In this phase, an irregular respiratory pattern progresses to apnoea. Ventilatory assistance is then initiated by the anaesthesiologist, usually through face mask and reservoir bag.[2,4]

Inhalation induction is a safe technique that is feasible and well accepted by patients, and is widely used in children or, exceptionally, in adults with difficult venous access. However, it is not a risk-free method. Complications such as coughing, laryngospasm, salivation, induction failure, and voluntary apnoea can increase the morbidity of the anaesthetic procedure.[3,5]

The effects of general anaesthesia on the respiratory system are well established, including a greater predisposition to airway obstruction and collapse. Its mechanisms are not fully understood, although some associations have been documented (relaxation of the ventilatory muscles, dose-effect relationship of the level of anaesthesia, and impairment of airway patency).[6,7] The loss of respiratory muscle tone resulting from induction of anaesthesia is related to collapse of the lower airways, reducing functional residual capacity (FRC).[7]

Another additive explanation for airway collapse is the pharmacological impact of the drugs used for sedation and hypnosis on the processes that control both the motor efferent pathways of the pharyngeal muscles and the mechanoreceptor afference.[6] Inhalational anaesthetics exacerbate dynamic airway collapse, especially in the soft palate, and this is directly associated with the dose of the drug administered. Sevoflurane reduces the inspiratory phasic activity of the genioglossus muscle without compromising its tonic activity. Even at sedative levels, sevoflurane modifies the dimensions of the airways, reducing their patency, especially during inspiration.[6]

Like inhaled drugs, venous anaesthetics also have an action on the respiratory system. Propofol reduces the cross-sectional area of the upper airway, a reduction that was shown to be at its maximum at the base of the tongue when sedative doses are used. Induction with this agent reduces the electromyographic activity of the genioglossus muscle.[6]

During the period of apnoea that follows the induction of general anaesthesia, oxygen (O_2) reserves are being consumed, which can result in hypoxemia.[8] Oxyhaemoglobin desaturation is faster in patients with reduced O_2 transport capacity, that is, in those with decreased CRF, partial pressure of oxygen (PaO_2), arterial oxygen content, and cardiac output, or increased oxygen consumption (VO_2).[9]

General anaesthesia is a major risk factor for mortality in paediatric surgical patients, and problems in airway management of patients with comorbidities also increase mortality risk in this population.[10] Paediatric patients, in turn, present desaturation episodes during induction more often (4%–10%).[5,8,11,12] Children submitted to a greater number of attempts of tracheal intubation, considered as having difficult

airways, are at a higher risk of desaturation that, if left untreated, naturally evolves to hypoxemia.[12] The occurrence of this during airway manipulation in children may be accompanied by even more severe complications such as cardiorespiratory arrest (CRA) and death.[11]

A drop in oxyhaemoglobin saturation to levels less than or equal to 95% induces changes in hemodynamic parameters such as the systolic index (ratio between the cardiac index [systolic volume × heart rate/body surface area] and heart rate), which signals a reduction in heart function.[13]

Data from a study conducted in a tertiary hospital over a 5-year period (12,158 surgeries) document respiratory events as responsible for 29% of perioperative cardiac arrest in children. This proportion increases to more than half (56%) when data related to anaesthesia in some way are included. All cardiac arrest (CA) attributed exclusively to anaesthesia occurred during induction.[14]

Another study (10,649 anaesthesias) performed by means of a questionnaire with data from a 6-year period indicated problems in airway management as the major cause of anaesthesia-related CA.[15]

Specific anatomical characteristics may contribute to the occurrence of hypoxemia in children in the perioperative period, such as: 1) proportionally large head and tongue; 2) hypertrophy of adenoids and tonsils; 3) smaller and narrower hypopharynx; 4) higher larynx at neck height; 5) inclined rather than right-angled vocal cords; 6) inverted U-shaped epiglottis; and 7) smaller airway radius compared with adults, which imposes more resistance to airflow according to Poiseuille's law ($R=8\eta L/\pi r^4$). All these factors imply daily challenges to airway manipulation, whether during

ventilation or intubation.[16] In infants, airway closure during induction of general anaesthesia occurs primarily in the anteroposterior direction, being uniform throughout the pharynx, which changes in older children in whom the epiglottis is the point of greater narrowing.[6]

The physiological characteristics of paediatric patients, such as lower FRC, higher VO_2, higher carbon dioxide (CO_2) production, and higher incidence of respiratory complications during the induction period when the O_2 supply is interrupted, also contribute to the drop in haemoglobin oxygen saturation.[9,11,16,17] Age also has a linear correlation with the duration of apnoea before oxyhaemoglobin desaturation, and the lower the patient's weight, the higher the incidence of severe episodes of the event.[8,11] The risks of obstruction and desaturation seem to be higher in children up to 3 years of age.[6]

One of the strategies used by the anaesthesiologist to prevent this complication is pre-oxygenation, which is the administering of O_2 above the levels usually breathed by the patient to increase his/her stores. This strategy allows the prolongation of the time period before oxyhaemoglobin desaturation.[9,11,18] In theoretical models, the physiological O_2 reserves (in lung, plasma and haemoglobin) can increase more than two and a half times when the inspired fraction of O_2 (FiO_2) is equal to 1. This increase occurs mainly at the expense of the alveolar fraction of the FRC, the main oxygen reservoir of the body.[8,17] It is also associated with VO_2 and cardiac output, and is responsible for the availability of O_2 to the patient.[8]

Preoxygenation, despite its benefits, may also contribute to the occurrence of oxyhaemoglobin desaturation. Microatelectasis and ventilation/perfusion (V/Q)

disturbance have been documented during induction of anaesthesia under different FiO_2. Alveolar recruitment manoeuvres and use of end-expiratory airway pressure (PEEP) can reverse and prevent, respectively, the occurrence of those events.[7,8.] Other preventive strategies, such as apnoeic oxygenation, have also been studied but it is not clear which technique is ideal.[19,20]

A variation of non-invasive ventilation, continuous positive airway pressure (CPAP), is a ventilatory mode in which the patient breathes spontaneously through a pressurized circuit.[21] In patients with obstructive sleep apnoea, in which its use is already well established, benefits such as improvement in sleepiness and disease severity, improvement in cardiovascular outcomes, and blood pressure reduction, and additional indirect effects such as improvement in insulin resistance in non-diabetics have been evidenced.[22-24]

From the ventilatory point of view, the benefits of CPAP are demonstrated by the improvement in alveolar gas exchange, minimization of atelectasis formation, and increase in both functional residual capacity and tidal volume.[21] Improvements in peripheral oxygen saturation, peak respiratory flow and reduction in both respiratory frequency and work of breathing have already been evidenced in patients during asthma crisis.[25]

In the paediatric population, CPAP has been widely studied in patients with bronchiolitis as an alternative to controlled mechanical ventilation due to its effects on small airways (alveolar opening, prevention of atelectasis, and increase in functional residual capacity).[26,27] A study with patients aged 3 months to 5 years with respiratory distress also showed a potential benefit by reducing respiratory rate.[28]

There is evidence that CPAP can be efficient in minimizing the deleterious effects of pre-oxygenation under high FiO_2 by maintaining pulmonary volume.[7] Its use in ventilation during induction of general anaesthesia still requires well-conducted studies to support its routine practice, but studies in adults have shown encouraging results.[29,30]

In patients without comorbidities and candidates for major surgery, the longer apnoea time before desaturation and higher PaO_2 values were significant in those who received CPAP during anaesthetic induction. The use of CPAP also reduced the time to return to normal baseline saturation values after apnea.[29]

The use of CPAP was also related to higher PaO_2 and lower arterial carbon dioxide pressure ($PaCO_2$) values in obese patients during induction of anaesthesia. There were no episodes of hypoxemia in those patients, unlike those who did not use the continuous positive pressure technique.[30]

The objective of the present study was to evaluate the effectiveness of CPAP during induction of anaesthesia in increasing the apnoea time until haemoglobin saturation falls to 95% in children undergoing general anaesthesia for elective surgery.

II. JUSTIFICATION

Anaesthetic induction is a critical period that requires special attention, mainly due to the period of apnoea that follows the use of hypnotic drugs. It is known that short periods of apnoea do not have major repercussions for the patient, but in difficult ventilation-intubation situations the occurrence of apnoea for a prolonged period can lead to oxyhaemoglobin desaturation.

Thus, evaluating the effectiveness of manoeuvres that can improve the ventilatory reserve is extremely relevant. This can lead to improvements in assistance and provide important evidence for the scientific community

After searching the LILACS, Scielo, Bireme, PubMed, and Cochrane Library databases, only a few studies were found in the adult population. We found no studies conducted in the paediatric population. Therefore, the present study might provide advances in the care of paediatric patients undergoing anaesthesia and increase the safety of anaesthetic practice.

According to the required sample size (calculated as 72 patients) and taking into account the number of surgeries performed per month in the paediatric surgical clinic, it will not be difficult to obtain the number of subjects needed to answer our research question. In addition, the researchers that will be involved in this study have *expertise* in paediatric anaesthesia and ventilatory conduction.

We would like to declare that this study will respect human rights, the principles of bioethics, resolution 466/2012 of the National Health Council, and the Declaration of

Helsinki. In addition, due to its novelty, the study will possibly be published in a relevant and high impact journal.

III. HYPOTHESIS

3.1 Primary hypothesis

- The use of CPAP in the pulmonary ventilation of preschool children during induction of general anaesthesia for elective surgery delays the occurrence of oxyhaemoglobin desaturation during the apnoea period.

3.2 Secondary hypotheses

- Pulse oxyhaemoglobin saturation values on pulse oximetry in apnoeic patients at similar periods during induction of anaesthesia are higher in those using CPAP.

- Time to recovery from normal oxyhaemoglobin saturation on pulse oximetry after a period of apnoea is shorter in patients using CPAP.

- The frequency of complications (laryngospasm, hypoxemia, bradycardia, cardiac arrest, and death) is lower in patients using CPAP.

IV. OBJECTIVES

4.1. General objective

To evaluate the effectiveness of CPAP during induction of anaesthesia in increasing the apnoea time until haemoglobin saturation falls to 95% in children undergoing general anaesthesia for elective surgery.

4.2. Specific objectives

In children undergoing general anaesthesia for elective surgery who will undergo CPAP or standard circular circuit ventilation during induction of anaesthesia, the following comparisons will be made:

PRIMARY ENDPOINT:

The time between the onset of apnoea and the fall in oxyhaemoglobin saturation to 95% in the different groups.

SECONDARY OUTCOMES:

1. Pulse oxyhaemoglobin saturation values on pulse oximetry during anaesthetic induction at different times in the different groups.

2. The time to recovery of oxyhaemoglobin saturation levels in pre-apnoea pulse oximetry in the different groups.

3. The frequency of complications (laryngospasm, hypoxemia, bradycardia, cardiac arrest, and death) in the different groups.

V. METHODS

5.1. Study design

This is a randomized, phase III, parallel clinical trial in paediatric patients undergoing elective surgery.

5.2. Study site

The study will be performed in the operating room of the Hospital das Clínicas of the Universidade Federal de Pernambuco (HC-UFPE).

The paediatric surgery service of HC-UFPE performs approximately 15–20 surgeries per week on an elective basis. The team consists of six surgeons and three resident physicians, who use a room in the operating suite for four shifts per week to perform procedures on children from birth to 18 years of age.

5.3. Period of the study

The study will be conducted during the period January 2018 to June 2018.

5.4. Study population

Paediatric preschool patients undergoing elective surgery in the surgical ward of the Hospital das Clínicas.

5.5. Samples

5.5.1. Sampling

A non-probability convenience sample will be obtained, composed of preschool children who will undergo general anaesthesia for elective surgeries, adhering to the inclusion and exclusion criteria of the study.

5.5.2. Sample size

The required sample size calculation was performed using the Openepi program, version 3.01 (Dean AG, Sullivan KM, Soe MM. OpenEpi: Open Source Epidemiologic Statistics for Public Health. www.OpenEpi.com, updated 2013/04/06, accessed 2017/07/11), using the difference of means. The first parameter used was the mean apnoea time that it took patients exposed to the intervention to reach an oxygen saturation on pulse oximetry of 95% (166±47 s).[20] The second parameter was the mean apnoea time it took patients not exposed to the intervention to achieve 95% oxygen saturation on pulse oximetry (131 ± 39 s).[20] Considering a significance level of 5% and a power of 90%, 64 patients (32 in each group) would be required. However, considering possible losses by post-randomization exclusion (approximately 10%), this number will be increased to 72 (36 in each group).

5.5.3. Procedure for randomization

The randomization table will be generated using the Random Software Allocation program. After randomization, opaque envelopes sequentially numbered from 1 to 72 will be prepared according to the random number table. The allocation concealment will be respected.

5.6. Criteria and procedures for selecting, attracting, and monitoring participants

5.6.1. Inclusion criteria

- Preschool children
- ASA I or II
- Children undergoing general anaesthesia for elective surgery

5.6.2. Exclusion criteria

- Pre-existing parenchymal lung disease

- Cyanotic children or children with oxyhaemoglobin saturation less than 95% before induction of anaesthesia
- Recent history (<4 w) or presence of upper respiratory tract infection

5.6.3. Procedure for recruiting and monitoring participants

The recruitment of participants will be carried out by a student who will not participate in data collection. He will be solely responsible for recruiting participants, applying the eligibility criteria using a checklist (Appendix 1), and requesting the signing of the Informed Consent Form (ICF). This step will take place at the entrance of the operating room where patients and companions leaving the outpatient clinic after weighing and measuring await surgery. Subsequently, the envelope containing the group to which the participant was allocated will be handed to the main researcher.

Allocation will be by sequentially numbered, otherwise identical, sealed envelopes, each containing a 2 inch by 2 inch piece of paper with a written code designating the intervention group or the comparison group. There will be no detectable differences in size or weight between the intervention group envelopes and the comparison group envelopes. The envelopes will be opaque and opened sequentially only after the information regarding the patients to whom they have been assigned is written on them. The opening of the envelope will occur before the patient enters the operating to complete study scenario set up.

The principal investigator will be responsible for the entire procedure, together with the anaesthesiologist responsible for the surgery. In turn, data collection will be performed by a student responsible only for data collection without knowledge of the

group to which the patient will be allocated (the CPAP system will be selected before the student enters the room for collection).

A flow chart (CONSORT) will be completed showing the progress through the phases of a two-group parallel intervention study (participant selection, intervention allocation, follow-up, and data analysis) (Figure 1).

All medical records of the participants involved in the study will be identified by means of self-adhesive labels containing the name of the study, record number, identification number of the patient, and the group to which he/she was allocated.

5.7. Flowchart outlining the participant intake and follow-up process

The flowchart outlining the process for recruiting and monitoring research participants is shown in Figure 1 below.

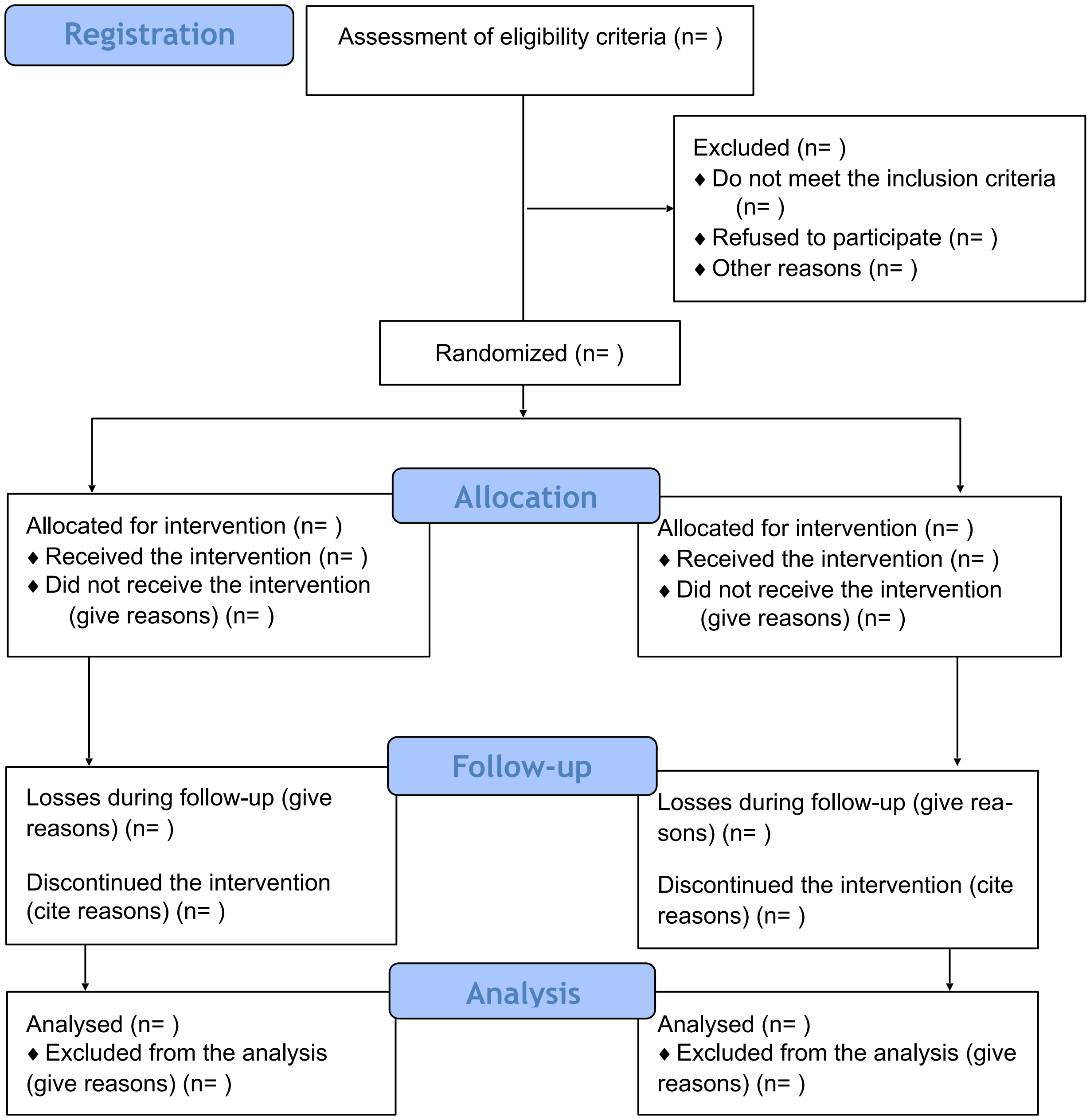

Figure 1. Flowchart outlining the participant intake and follow-up process

5.8. Terms, variables, and concepts

5.8.1. Control variables (for sample characterisation)

- Age
- Weight
- Height
- Sex
- Physical state according to ASA
- Type of surgery

5.8.2. Independent variable

- Use of CPAP

5.8.3. Dependent variables

- Time between the onset of apnoea and the drop in oxyhaemoglobin saturation to 95% (Time 1).
- Oxyhaemoglobin saturation on pulse oximetry during induction of anaesthesia at different time points.
- Frequency of complications: laryngospasm, hypoxemia, bradycardia, cardiac arrest, and death.
- Time to recovery of oxyhaemoglobin saturation levels on pre-apnoea pulse oximetry (Time 2).

5.8.4. Definition of terms and variables

Term/Variable	Definition	Categorization
CPAP	Ventilatory mode in which the patient breathes spontaneously through a pressurized circuit against a threshold resistor that maintains a specific pressure during both inspiration and expiration[31]	Independent variable
Central apnoea	Transient cessation of breathing for any duration usually accompanied by bradycardia and/or cyanosis[32]	-
Pulmonary ventilation	Total volume of gas inspired or expired in the unit of time[33]	-
Pre-school	Child whose age range is 2–6 years old	-

Oxyhaemoglobin	Compound formed by the combination of haemoglobin and oxygen in which the latter binds directly to the iron without causing a change from the ferrous to the ferric state	-
Pulse oximetry	Determination of blood oxyhaemoglobin saturation through electrodes attached to some translucent part of the body (finger, ear lobe, skin fold)[34]	-
Laryngospasm	Respiratory complication resulting from reflex glottis closure, most common in children during superficial anesthesia[35]	Dependent variable, qualitative, nominal, dichotomous, having categories of yes or no

General anaesthesia	Transient stage of unconsciousness induced by drugs through their actions on molecular receptors[1]	-
Parenchymal lung disease	Diverse group of lung diseases characterized initially by inflammation of the alveoli that extends into the interstitium, leading to diffuse pulmonary fibrosis	-
Cyanosis	Bluish or purple coloration of the skin and mucous membranes due to increased deoxygenated haemoglobin in the blood or a structural defect in the haemoglobin molecule	-

Upper respiratory tract infection	Invasion of the upper respiratory tract of the host by a pathogen, usually a virus or bacterium. The main diseases classified in this group are viral rhinopharyngitis, acute sinusitis, acute streptococcal pharyngamoidalitis, and acute viral laryngitis. Acute viral rhinopharyngitis is the most common representative of this group and is characterized by sore throat, runny nose, nasal obstruction, sneezing, dry cough, and fever of variable intensity[36]	-

Time between the onset of apnoea and the drop in 95% oxyhaemoglobin saturation	-	Dependent variable, quantitative, numerical, continuous, measured in seconds
Oxyhaemoglobin saturation in pulse oximetry during induction of anaesthesia at different times	-	Dependent variable, quantitative, numerical, discrete, measured in percentage points
Time to recovery from oxyhaemoglobin saturation levels on pre-apnoea pulse oximetry	-	Dependent variable, quantitative, numerical, continuous, measured in seconds
Age	-	Quantitative, numerical, continuous variable, measured in completed months of life
Weight	-	Quantitative, numerical, continuous variable, measured in grams
Height	-	Quantitative, numerical, continuous variable, measured in centimetres

Sex	-	Qualitative, nominal, dichotomous variable, having categories of male and female
Physical status according to the American Society of Anesthesiology (ASA)	Physical status of the patient based on the presence/absence of disease in patients or on non-pathological physiological changes, as well as on the quality of control of morbidities[37]	Ordinal variable whose categories are numbered in Roman numerals from I to VI, with I indicating the absence of systemic disease, and II indicating controlled systemic disease[38]

.

5.9. Procedures, tests, techniques, and examinations

5.9.1. Delivery of anaesthesia

Patients selected for the study will be admitted to the operating room and will receive usual monitoring (cardioscope, oximeter, non-invasive blood pressure and capnography).

Inhalation induction will use sevoflurane 8% and inspired oxygen fraction of 60.5% under a fresh gas flow of 4 L/min (2 L O_2 and 2 L compressed air) until loss of the eyelid reflex. The concentration of the anaesthetic will then be reduced to 4%. A face mask attached to the anaesthesia machine will be used, fixed to the patient using an elastic band.

After adequate ventilation is verified by correct positioning of the face mask and capnography curve present, peripheral venous access will be obtained with a 20, 22, or 24 G venous catheter for hydration and infusion of propofol at a dose of 3.5 mg/kg to induce apnoea in patients of both groups.

5.9.2. Performing CPAP

The patients will be submitted to the technique described in the envelopes delivered at the entrance of the operating room immediately after monitoring.

In the CPAP group, this will be applied by the anaesthesia machine (Dräger Fabius GS) using a circular system. This system consists of two corrugated tubes coupled at one end to a Y-shaped piece connected to the patient's face mask, and at the other end to the anaesthesia equipment, which, in addition to providing the flow of fresh gases, has a CO_2 absorber called soda-lime. Besides enabling a circular system by removing CO_2 from the air supplied to the patient, this heats and humidifies the gas mixture. A pressure-limiting valve (pop-off valve), which prevents gas loss through the patient-apparatus system when closed, is an integral part of the anaesthesia equipment. It has several markings (0–70 cmH_2O), is manually manipulated, and can be opened (0 cmH_2O), i.e. no pressure is delivered to the patient's airway, or closed. In the closed position, a continuous positive pressure is delivered to the patient's airway. The pressure that will be used in this group will be 10 cmH_2O.

In the Open System group, the system will remain with the valve in the open position, i.e. 0 cmH_2O.

In both groups, patients will ventilate spontaneously from the outset with the technique defined at the time of allocation.

After the onset of apnoea, the time will be recorded and periodically (every 10 s) the oxyhaemoglobin saturation will be registered through the pulse oximeter for a maximum time limit of 5 min in order to register a minimum saturation of 95% in both groups.

During all anaesthetic procedures, an anaesthesiologist will accompany the patient and will be responsible for them once the study is over.

5.10. Procedures for data collection

5.10.1. Data collection instrument

Data will be collected using a standardized, pre-coded form for data entry into the computer (Appendix 3). The information for categorical variables will be pre-coded and continuous variables will be expressed as their own numerical value and only at the time of analysis will the results of some of these be categorized.

These forms will be duly stored in specific file folders, before and after data entry and analysis, under the responsibility of the researcher himself, who will fill them in at different times, before, during, and after the surgical procedure.

5.10.2. Data collection

Data will be collected by an independent researcher who will be present in the operating room. He/she will fill in the form with the patient's identification data and study variables and will not participate in the anaesthetic procedure to be performed.

The time from the moment respiratory movements cease and the capnography curve decays will be timed. The measurement will take place until the pulse oximetry registers the value of 95%. Assisted ventilation will then be instituted (in the Open System group, the pop-off valve will be manually closed at a value of 10 cmH_2O). At

this point, the time taken until the pulse oximetry reading of 100% or the value obtained immediately before the onset of apnoea will be recorded.

5.11. Data processing and analysis

5.11.1. Data processing

Date entry into the specific database created in the Excel program will be performed twice, at different times and by different people. Inconsistencies due to typing errors will be collated to a list and evaluated by the researcher himself.

If inconsistencies or missing data are found when the list is reviewed, the corresponding archived forms will be consulted, according to the registration number of the patients.

Once all the data have been entered in the database, the final revision will be rendered and the data missing from the above-mentioned processes will be added. The final database that is created will then be used for statistical analysis using the STATA program. This will be subjected to consistency and information cleaning tests, and backup copies will be generated.

5.11.2. Data analysis

Data analysis will be performed by the researcher and the statistician in charge (supervised by her advisor), using the STATA program.

Descriptive statistical analysis will be performed through measures of central tendency and dispersion for quantitative variables and through frequency distribution for qualitative variables. For comparison of paired samples, the non-parametric Wilcoxon's T-test will be used.

The Student's t-test for two independent samples will be used, including the Kolmogorov-Smirnoff normality test for each sample, and in the case of non-normality, the nonparametric Mann-Whitney U test will be used. Analysis of variance (ANOVA) for repeated units will be used for quantitative variables according to the time of their performance, if they present a normal distribution, or Wilcoxon’s test will be used if normality is not present. For categorical variables, the Fisher's exact test or the Chi-squared test will be used when necessary.

The Relative Risk and its confidence interval at 95% will also be calculated, in addition to the number needed to treat (NNT) or the number needed to arm (NNH) when relevant.

5.12. Ethical aspects

The research will respect human rights and bioethical principles (Autonomy, Non-Maleficence, Beneficence, Justice and Equity). Confidentiality in terms of the collection and storage of collected data will be respected.

The terms of resolution No. 466 of 12 December 2012 of the National Health Council for research in humans will be followed, as well as the principles of the Declaration of Helsinki. In addition, the study proposal will be submitted to the Research Ethics Committee (REC) of the proposing institution. Data will only be collected after such submission and approval by the REC and children will only be included in the study after their guardians sign the ICF (Appendix 2).

The study will be registered at *ClinicalTrials.gov* and will not pose risks or additional discomforts besides those inherent to the anaesthetic-surgical procedure itself, and will cause no adverse effects that contraindicate the use of CPAP during

induction have been previously reported in the literature. On the contrary, the few existing studies to date have reported respiratory improvement with the use of this intervention. All research procedures will be carried out by professionals trained in both paediatric anaesthesia and CPAP.

The Terms of Free and Informed Consent (TCLE) form (Appendix 2) will provide information to the caregivers regarding the advantages and disadvantages of using the two techniques. It will emphasize that no procedures that are no longer routinely used by the team will be performed, and it will include the right to refuse to participate in the study, as well as the guarantee of assistance to those who do not accept, without compensation from the institution or from the researchers. The researchers will be committed to publishing the study, regardless of the results obtained.

5.13. Conflicts of interest

This research will be free of conflicts of interest, private or institutional. There will be no funding from the pharmaceutical industry or representatives of any research equipment used in the study.

VI.

Table 1. Variables for characterization of the sample of preschoolers undergoing general anesthesia for elective pediatric surgery at HC-UFPE in 2018

**Variable**	**Group C (n)**	**Group A (n)**
**Age, months (mean±SD)**		

Table 1. Variables for characterization of the sample of preschoolers undergoing general anesthesia for elective pediatric surgery at HC-UFPE in 2018

Variable	Group C (n)	Group A (n)
**Weight, grams (mean±SD)**		
**Height, cm (mean±SD)**		
**Sex (n/%)**		

Table 1. Variables for characterization of the sample of preschoolers undergoing general anesthesia for elective pediatric surgery at HC-UFPE in 2018

**Variable**	**Group C (n)**	**Group A (n)**
Male		
Female		
**ASA (n/%)**		
I		
II		
**Pediatric surgery (n/%)**		
Postectomy		
Umbilical herniorrhaphy		
Inguinal herniorrhaphy		
Hypospadias correction		
Orchidopexy		

SD - standard deviation, Group C - CPAP group, Group A - Open System group, ASA - physical status according to the American Society of Anesthesiology

OUTTURN PLAN

Table 2. Times between the onset of apnoea and 95% saturation and recovery of pre-apnoea saturation levels in preschool children undergoing general anaesthesia for elective paediatric surgery at HC-UFPE in 2018

**Variable**	**Group C (n)**	**Group A (n)**	**Range**	**95%CI**	*p*
**Time (in seconds) between apnoea onset and 95% saturation (mean±SD)**					*
**Time (in seconds) to recovery from pre-apnoea saturation levels (mean±SD)**					*

Student's *t*-test, SD - standard deviation, Group C - CPAP group, Group A - Open System group

Table 3. Oxygen saturation during anaesthetic induction at different times in preschool children undergoing general anaesthesia for elective paediatric surgery at HC-UFPE in 2018

Variable	Group C (n)	Group A (n)	Range	95% CI	*p*
**Oxygen saturation during induction of anaesthesia (mean±SD)**					*
10”					
20”					
30”					
40”					
50”					
60”					
70”					
80”					
90”					
100”					
110”					
120”					
130”					
140”					
150”					

Student's *t*-test, SD - standard deviation, Group C - CPAP group, Group A - Open System group

Table 4. Frequency of complications during induction in preschool children undergoing general anaesthesia for elective paediatric surgery at HC-UFPE in 2018

**Variable**	**Group C (n)**	**Group A (n)**	**RR**	**95% CI**	*p*
**Complications (n/%)**					*
Laryngospasm					**
Hypoxemia					**
Bradycardia					**
Cardiac arrest					**
Death					**

* *z* test, ** Pearson's Chi-squared test, SD - standard deviation, Group C - CPAP group, Group A - Open System group

VII. BUDGET

Expenses will be paid by the authors of the study. The medications and materials used for anaesthesia are currently used in the Anaesthesiology Service to perform routine procedures in the operating room of HC-UFPE.

ITEM	HUMAN RESOURCE / MATERIAL	UNIT PRICE (R$)	TOTAL PRICE (R$)
01	Teacher (Service Coordinator)	Free of charge	0
01	Statistician	R$2,000.00	R$2,000.00
500	White sulphite paper, 75 g/m², A4	R$21.90	R$21.90
03	Epson XP-401 black printer cartridges	R$30,00	R$90,00
200	Photocopies	R$0.20	R$40.00
01	Office supplies (pens, clipboards, highlighter, stapler, staples)	R$30.00	R$30.00
01	Proof-reader	R$350.00	R$350.00
01	Translator	R$3,000.00	R$3,000.00
01	Publication fee	R$5,000.00	R$5,000.00
TOTAL			R$10,501.90

____

Pesquisador

VIII. SCHEDULE

	Sep/17	Oct/17	Nov/17	Dec/17	Jan/18	Feb/18	Mar/18	Apr/18	May/18	Jun/18	Jul/18	Aug/18	Sep/18	Oct/18	Nov/18	Dec/18	Jan/19	Feb/19
Preparation of the project	X																	
Literature review	X	X	X	X	X	X	X	X	X	X	X	X	X	X	X	X	X	
Presentation of the project		X																
Preparation of the team and testing of instrumentation		X	X															
Data collection				X	X	X	X	X	X	X								
Data revision and correction (forms)										X								
Data entry											X							
Cleaning and consistency testing												X						
Tabulation and analysis of data													X					
Review of data analysis													X					
Writing of dissertation													X	X				
Revision of the dissertation														X				
Drafting of the article															X	X	X	
Dissertation Defence																		X
Article delivery																		X

Data collection will only be initiated after project approval by the REC.

IX. REFERENCES

1. Århem P, Klement G, Nilsson J. Mechanisms of Anesthesia: Towards Integrating Network, Cellular, and Molecular Level Modeling. *Neuropsychopharmacology*. 2003;28(S1):S40-S47.
2. Schwartz RS, Brown EN, Lydic R, Schiff ND. General Anesthesia, Sleep, and Coma. *N Engl J Med*. 2010;363(27):2638-2650.
3. Boonmak P, Boonmak S, Pattanittum P. High initial concentration versus low initial concentration sevoflurane for inhalational induction of anaesthesia. Boonmak P, ed. *Cochrane database Syst Rev*. 2012;2016(9):CD006837.
4. Park JH, Kim JY, Lee JM, Kim YH, Jeong HW, Kil HK. Manual vs. pressure-controlled facemask ventilation for anaesthetic induction in paralysed children: a randomised controlled trial. *Acta Anaesthesiol Scand*. 2016;60(8):1075-1083.
5. von Ungern-Sternberg BS, Boda K, Chambers NA, et al. Risk assessment for respiratory complications in paediatric anaesthesia: a prospective cohort study. *Lancet*. 2010;376(9743):773-783.
6. Ehsan Z, Mahmoud M, Shott SR, Amin RS, Ishman SL. The effects of Anesthesia and opioids on the upper airway: A systematic review. *Laryngoscope*. 2016;126(1):270-284.
7. Hedenstierna G, Edmark L. Effects of anaesthesia on the respiratory system. *Best Pract Res Clin Anaesthesiol*. 2015;29(3):273-284.
8. Bouroche G, Bourgain JL. Preoxygenation and general anaesthesia: a review. *Minerva Anestesiol*. 2015;81(8):910-920.

9. Nimmagadda U, Salem MR, Crystal GJ. Preoxygenation: Physiologic Basis, Benefits, and Potential Risks. *Anesth Analg*. 2017;124(2):507-517.

10. Gonzalez L, Pignaton W, Kusano P, Modolo N, Braz J, Braz L. Anesthesia-related mortality in pediatric patients: a systematic review. *Clinics*. 2012;67(4):381-387.

11. Fiadjoe JE, Nishisaki A, Jagannathan N, et al. Airway management complications in children with difficult tracheal intubation from the Pediatric Difficult Intubation (PeDI) registry: a prospective cohort analysis. *Lancet Respir Med*. 2016;4(1):37-48.

12. Long E, Sabato S, Babl FE. Endotracheal intubation in the pediatric emergency department. Anderson B, ed. *Pediatr Anesth*. 2014;24(12):1204-1211.

13. King MR, Anderson TA, Sui J, He G, Poon KYT, Coté CJ. Age-related incidence of desaturation events and the cardiac responses on stroke index, cardiac index, and heart rate measured by continuous bioimpedance noninvasive cardiac output monitoring in infants and children undergoing general anesthesia. *J Clin Anesth*. 2016;32:181-188.

14. Bharti N, Batra YK, Kaur H. Paediatric perioperative cardiac arrest and its mortality: database of a 60-month period from a tertiary care paediatric centre. *Eur J Anaesthesiol*. 2009;26(6):490-495.

15. Gonzalez LP, Braz JRC, Módolo MP, de Carvalho LR, Módolo NSP, Braz LG. Pediatric Perioperative Cardiac Arrest and Mortality. *Pediatr Crit Care Med*. 2014;15(9):878-884.

16. Bhananker S, Harless J, Ramaiah R. Pediatric airway management. *Int J Crit Illn Inj Sci*. 2014;4(1):65.

17. Chiron B, Mas C, Ferrandière M, et al. Standard preoxygenation vs two techniques in children. *Paediatr Anaesth*. 2007;17(10):963-967.

18. De Jong A, Futier E, Millot A, et al. How to preoxygenate in operative room: healthy subjects and situations "at risk". *Ann Fr Anesth Reanim*. 2014;33(7-8):457-461.

19. Humphreys S, Lee-Archer P, Reyne G, Long D, Williams T, Schibler A. Transnasal humidified rapid-insufflation ventilatory exchange (THRIVE) in children: a randomized controlled trial. *Br J Anaesth*. 2017;118(2):232-238.

20. Windpassinger M, Plattner O, Gemeiner J, et al. Pharyngeal Oxygen Insufflation During AirTraq Laryngoscopy Slows Arterial Desaturation in Infants and Small Children. *Anesth Analg*. 2016;122(4):1153-1157.

21. Faria DAS, da Silva EMK, Atallah ÁN, Vital FMR. Noninvasive positive pressure ventilation for acute respiratory failure following upper abdominal surgery. Vital FM, ed. *Cochrane database Syst Rev*. 2015;(10):CD009134.

22. Bratton DJ, Stradling JR, Barbé F, Kohler M. Effect of CPAP on blood pressure in patients with minimally symptomatic obstructive sleep apnoea: a meta-analysis using individual patient data from four randomised controlled trials. *Thorax*. 2014;69(12):1128-1135.

23. Wang J, Yu W, Gao M, et al. Continuous positive airway pressure treatment reduces cardiovascular death and non-fatal cardiovascular events in patients with

obstructive sleep apnea: A meta-analysis of 11 studies. *Int J Cardiol*. 2015;191:128-131.

24. Iftikhar IH, Khan MF, Das A, Magalang UJ. Meta-analysis: Continuous Positive Airway Pressure Improves Insulin Resistance in Patients with Sleep Apnea without Diabetes. *Ann Am Thorac Soc*. 2013;10(2):115-120.

25. de Freitas Dantas Gomes EL, Costa D, Germano SM, Borges PV, Sampaio LMM. Effects of CPAP on clinical variables and autonomic modulation in children during an asthma attack. *Respir Physiol Neurobiol*. 2013;188(1):66-70.

26. Jat KR, Mathew JL. Continuous positive airway pressure (CPAP) for acute bronchiolitis in children. Jat KR, ed. *Cochrane database Syst Rev.* 2015;1:CD010473.

27. Sinha IP, McBride AKS, Smith R, Fernandes RM. CPAP and High-Flow Nasal Cannula Oxygen in Bronchiolitis. *Chest*. 2015;148(3):810-823.

28. Wilson PT, Morris MC, Biagas K V, Otupiri E, Moresky RT. A randomized clinical trial evaluating nasal continuous positive airway pressure for acute respiratory distress in a developing country. *J Pediatr*. 2013;162(5):988-992.

29. Sreejit M, Ramkumar V. Effect of positive airway pressure during pre-oxygenation and induction of anaesthesia upon safe duration of apnoea. *Indian J Anaesth*. 2015;59(4):216.

30. Harbut P, Gozdzik W, Stjernfält E, Marsk R, Hesselvik JF. Continuous positive airway pressure/pressure support pre-oxygenation of morbidly obese patients. *Acta Anaesthesiol Scand*. 2014;58(6):675-680.

31. Squadrone V, Coha M, Cerutti E, et al. Continuous positive airway pressure for treatment of postoperative hypoxemia: a randomized controlled trial. *JAMA*. 2005;293(5):589-595.

32. Pradhapan P, Swaminathan M, Salila Vijayalal Mohan HK, Sriraam N. Identification of apnea during respiratory monitoring using support vector machine classifier: a pilot study. *J Clin Monit Comput*. 2013;27(2):179-185.

33. Simon BA, Kaczka DW, Bankier AA, Parraga G. What can computed tomography and magnetic resonance imaging tell us about ventilation? *J Appl Physiol*. 2012;113(4):647-657.

34. Jubran A. Pulse oximetry. *Crit Care*. 2015;19(1):272.

35. Roy WL, Lerman J. Laryngospasm in paediatric anaesthesia. *Can J Anaesth*. 1988;35(1):93-98.

36. Pitrez PMC, Pitrez JLB. Acute upper respiratory tract infections: outpatient diagnosis and treatment. *J Pediatr* (Rio J). 2003;79(suppl.1):77-86. http://dx.doi.org/10.1590/S0021-75572003000700009.

37. Saklad M. Grading of patients for surgical procedures. *Anesthesiology*. 1941;2(3):281-284.

38. ASA Physical Status Classification System. https://www.asahq.org/resources/clinical-information/asa-physical-status-classification-system. Published 2014.

APPENDIX 1 - CHECKLIST

DATE

Name: ....

INCLUSION CRITERIA

	Y e<br>s	No
ASA I or II		
Preschool children		
Children undergoing general anaesthesia for elective paedia-tric surgery		

EXCLUSION CRITERIA

	Yes	No
Pre-existing parenchymal lung disease		
Cyanotic children or children with oxyhaemoglobin saturation less than 95% before induction of anaesthesia		
Recent history (<4 weeks) or history of upper respiratory tract infecti-on		

Eligible: Yes ( ) No ( )

Responsible person has signed the informed consent form: Yes ( ) No ( )

APPENDIX 2 - INFORMED CONSENT FORM

Prof. Fernando Figueira Institute of Integral Medicine/Postgraduate School of Maternal and Child Health

INFORMED CONSENT FORM

We request your authorization to invite your child {or the minor under your responsibility} ________________ to participate as a volunteer in the research study entitled 'CONTINUOUS POSITIVE PRESSURE IN THE AIRWAYS DURING THE INDUCTION OF GENERAL ANAESTHESIA FOR ELECTRIC PAEDIATRIC SURGERY: RANDOMIZED CLINICAL TRIAL'. In order for you to decide whether or not to participate, you need to know the benefits, risks, and consequences of participation.

This document is called an Informed Consent Form (ICF) and has this name because you should only participate in the research after you have read and understood this statement. Read the information carefully and talk to the responsible researcher and research team about any questions you may have. If there is a word or term that you do not understand, talk to the person responsible for obtaining this consent for further clarification. If you prefer, talk to your family, friends, and medical staff before making a decision. After receiving all the information, you may provide your consent, initialling and/or signing the two copies, one for the responsible researcher and the other for the research participant.

If you do not agree, neither you nor the child under your care will be penalized, and you may withdraw consent at any time, also without penalty.

This is a research project under the responsibility of the researcher Jayme

Marques dos Santos Neto, who is an anaesthesiologist studying a technique to help children's breathing during surgery. The researcher's address is: Avenida Boa Viagem, 306 apto. 701, Pina, Recife-PE CEP 51011-000; telephone (81) 996212977. Dr. Jayme is working on his Master's thesis and is being supervised by Dr. Flávia Augusta de Orange Lins da Fonseca e Silva who is also an anaesthesiologist. Telephone 81994197979, e-mail orangeflavia@gmail.com

INFORMATION ABOUT THE RESEARCH:

Your child is being invited to participate in research that will study the effect of a technique to help breathing during the onset of anaesthesia. We call this technique continuous positive airway pressure (CPAP).

Children need to receive general anaesthesia in order to be operated on. To receive this, they breathe wearing a silicone or plastic mask that is connected to the anaesthetic machine. The gas that comes from the anaesthesia machine contains the medicine that makes the child sleep. This technique is popularly known as "sniffing", but it is actually General Anaesthesia, and it is commonly used in almost all anaesthesia in children. CPAP is done in the same way. The only difference to habitual ventilation is that in CPAP the anaesthesia machine provides a pressure that can help (that's what we want to find out) children to breathe better.

Two groups will be formed in which in one group of children will receive CPAP and the other group of children will receive regular ventilation. We do not know which group your child will be in. Their participation is not mandatory. The goal of this project is to find out if CPAP at the beginning of anaesthesia improves the child's safety and if the oxygen in the child's blood stays at normal levels for longer.

The participation of your child ends as soon as she returns to the initial condition in the study when his/her surgery will then be performed. We want to make it clear that this is not a new method; it has been used before, and is considered a safe technique. In previous studies, no side effects or complications were verified.

It is expected that, as a result of this study, the use of CPAP at the beginning of anaesthesia can be increasingly encouraged, improving the quality of care in anaesthesia. All anaesthesia will involve the participation of the anaesthesiologist responsible for the surgery in addition to the researcher who will be present during the entire period of the study, thus increasing the vigilance of procedures performed on the study participants.

The occurrence of undesirable effects is possible in any research study, such as embarrassment when signing this form, despite all possible care, and can happen through no fault of your own or the researchers. If your child suffers any undesirable effects or harm associated with participation in this study, immediate and full professional assistance will be provided.

The possible advantages for your child are more oxygen in their blood, less chance of problems at the beginning of anaesthesia, increased safety time for them in case any problems occur at the beginning of anaesthesia, and faster recovery if they stop breathing.

Information obtained by this research will remain confidential and be disclosed only during scientific conferences or in scientific publications. The volunteers will be identified only to the researchers responsible for the study and their details will remain otherwise confidential. The data collected through forms will be stored in file folders,

under the responsibility of the researcher, at the address supplied above, for a period of at least 5 years.

You will not pay anything or receive any payment for participating in this research because it is voluntary, but compensation is guaranteed in the case of damages demonstrably resulting from participation in this research, according to judicial or extra-judicial decision. If necessary, expenses incurred by participation will be assumed by the researchers (reimbursement for transportation and food).

In case of doubts relating to the ethical aspects of this study, you may consult the Ethics Committee for Research Involving Human Beings of IMIP at this address: **Rua dos Coelhos, nº 300, Boa Vista. IMIP's Research Board, Orlando Onofre Administrative Building, 1st floor tel: 2122-4756 - Email:** comitedeetica@imip.org.br. CEP/IMIP opening hours are Monday to Friday, from 07:00 to 11:30 am (morning) and from 1:30 to 4:00 pm (afternoon).

____

Signature of Researcher (a)

CONSENT OF THE PERSON RESPONSIBLE FOR THE PARTICIPATION OF THE VOLUNTEER

I, ______________________________________, CPF_________________, the undersigned, responsible for ______________________________, authorize his/her participation in the study 'CONTINUOUS POSITIVE PRESSURE IN THE AIRWAY DURING INDUCTION OF GENERAL ANAESTHESIA FOR ELECTRIC PAEDIATRIC SURGERY: RANDOMIZED CLINICAL TRIAL', as a volunteer. I have

been duly clearly informed by the researcher regarding the research and the procedures involved, as well as the possible risks and benefits resulting from his/her participation. I was assured that I may withdraw my consent at any time without this leading to any penalty or interruption of the follow-up/assistance/treatment for me or for the minor in question.

Place and date ___________________

Signature of the responsible person: ___________________________

We witnessed the request for consent, explanation regarding the research, and acceptance of the subject to participate. Two witnesses (not connected with the **research** team):

Name:	Name:
Signature:	Signature:

APPENDIX 3 - FORM

FORM Nº

GROUP:

DATE

<u>Patient characteristics</u>

Name: ....

Date of admission: / / ; Registration:

Date of birth : / / ; ASA: I II

Age (months): ; Weight (g): ; Height (cm): ; Sex: M ( ) F ( )

Surgery:

Pulse oximetry value at the onset of apnoea:

Time between the onset of apnoea and the drop in 95% oxyhaemoglobin saturation (seconds):

Pulse oximetry values

10”: ; 20”: ; 30”: ; 40”: ; 50”: ; 60”: ; 70”: ; 80”: ; 90”: ; 100”: ; 110”: ; 120”: ; 130”: ; 140”: ; 150”: .

Time to recovery from oxyhaemoglobin saturation levels in pre-apnoea pulse oximetry (seconds):

Complications: Laryngospasm ( ) Hypoxemia ( ) Bradycardia ( ) Cardiorespiratory arrest ( ) Death ( )
