# Supplementary material for: Continuous positive airway pressure to reduce the risk of early peripheral oxygen desaturation after onset of apnoea in children: A double-blind randomised controlled trial
Source: PLoS One. 2021 Oct 1;16(10):e0256950. doi: 10.1371/journal.pone.0256950 (PMC8486132; doi:10.1371/journal.pone.0256950)
Supplement: Attachment — Submitted filename: Response to Reviewers.docx [file pone.0256950.s015.docx]

1. Please ensure that your manuscript meets PLOS ONE's style requirements, including those for file naming. Adjusted in the manuscript.
2. We suggest you thoroughly copyedit your manuscript for language usage, spelling, and grammar. If you do not know anyone who can help you do this, you may wish to consider employing a professional scientific editing service. The original manuscript with tracked changes has been sent to Editage website ([www.editage.com](http://www.editage.com/)). Some changes in the text has been made before and after the edition. Due to this, the Revised Manuscript with Track Changes is different from the Manuscript.
3. Please remove any funding-related text from the manuscript and let us know how you would like to update your Funding Statement. Please include your amended statements within your cover letter. Funding-related text removed from the manuscript. JMSN received a scholarship provided by Coordenação de Aperfeiçoamento de Pessoal de Nível Superior (CAPES) (<https://www.gov.br/capes/pt-br>). The funders had no role in study design, data collection and analysis, decision to publish, or preparation of the manuscript.
4. Please include a separate caption for each figure in your manuscript. Adjusted in the manuscript.
5. Could the authors elaborate on the stability and the severity of SpO2 in the 30-second intervals within the CPAP group? Namely, this analysis (and hence result) could be related back to the limitation of the spontaneous ventilation and apnea situation during their anesthesia. Thank you. The curve of repeated measures shows a less pronounced drop compared to that of the control group. We can theorize that this is due to greater alveolar stability with maintenance of the opening of these and preservation of the functional residual capacity, the main body oxygen supply. During situations of limited ventilation and apnoea, the continuous flow of oxygen to the alveoli could maintain the levels of partial pressure of the gas in the blood at adequate levels for a longer time, increasing the safety margin for the patient and a greater time for the anesthesiologist to define strategies to face the situation.
6. Could the authors discuss the applicability of this approach to children with developmental disabilities undergoing surgery. Thank you for the opportunity. Oxyhemoglobin desaturation is faster in patients with reduced O_2_ transport capacity, that is, in those with reduced FRC. General anesthesia appears as a major risk factor for mortality in pediatric surgical patients, as well as problems with airway management in patients with comorbidities seems to add life-threatening to this population. In paediatric patients, lung volume and capacity are directly related to weight, height, and age, i.e. the younger and smaller the child, the lower the FRC, this risk being apparently greater in children under three years of age. We can speculate, therefore, that children with developmental disabilities may be at increased risk of hypoxemia during the period of apnea since they have in theory impaired pulmonary oxygen reserves. This work did not direct its scope to the investigation of these patients, however, it is possible to imagine that in a research that applies CPAP to these children under the conditions described here, documenting the effect of this ventilatory mode on FRC, the outcomes could be favorable in relation to the reduction of risk desaturation and prolonging a safe apnea time.
7. The primary outcome is not clearly described. At first reading, it seems to be the risk of SpO_2_ of 95% within a 5 minute period (indeed the sample size calculation is that for estimating a risk/proportion). However, reading the manuscript it is repeatedly referred to as hazard of the outcome, implying that the outcome is a time-to-event. The authors need to clarify this. Thank you. We tried to clarify it in the text with some modifications.
8. The units and resolution of measurement of the outcome T1 is not clear: was it measured down to the second or was. it also measured in 30 second increments? Thank you. The T1 was measured down to the second. Once its record was 95%, we stopped the clock and registered the exact second in which it occurred. During the time elapsed from the start of the apnoea and the 95% threshold, we chose the reference points 10, 20, 30, 40 seconds and so on to record the pulse oximetry value at that moments. Therefore, we recorded SpO2 values every 10 seconds on the data collection form of each patient. For graphic recording purposes, we group these measurements at a 30-second intervals.
9. If the primary outcome is a risk/proportion, then the sample size calculation is not sufficiently described as it does not include the 'baseline' risk of the outcome; this should be included along with a citation to support it. It is also not clear whether the 50% risk reduction proposed is an absolute or relative reduction. However, if the outcome is time-to-event, then the sample size calculation is incorrect, because other than including the hazard of the outcome in the 'baseline' group, it must also include some determination of the expected number of events. The sample size was calculated using Schoenfeld’s procedure with a sample of at least 33 patients in each randomized group. The study would have a 80% power to detect a 50% reduction in the hazard of the CPAP group, allowing only a 5% chance of a type I error at a two-sided significance test. However, after your observation, we reviewed this calculation and in fact we did not account for the percentage of censored values in the control group. Thus, using the Schoenfeld method, the expected number of events was 66. But supposing that, in the control group, 5% of patients reach the end of the follow-up time with SPO2> 95%, the sample size of each group would be 38 with a significance level of 5% and a power of 80%. On the other hand, if we change only the hazard ratio to 0.45, the sample size for each group would be 30 and the expected number of events would be 50. Please, would be so kind as to help us to find a way out of this issue?
10. For the descriptive analysis the authors should follow standard reporting for randomised trials, where the mean and SD for continuous baseline variables or counts and proportions for categorical baseline variables be presented by treatment arm without statistical tests comparing the arms. Thank you. Altered in the manuscript
11. If the primary outcome is a risk within a defined time period (5 minutes) therefore describing it as a hazard and estimating hazard ratios comparing the treatment arms for this outcome is not appropriate. It is however appropriate for the secondary outcome T1 (and perhaps T2). Thank you. We tried to clarify it in the text with some modifications.
12. The results for the three outcomes should be tabulated in the standard reporting format for a clinical trial i.e. including the event numbers and denominators per group, risks/hazards/rates as appropriate and their ratios, confidence intervals and p-values. Added Table 2.
13. Please indicate whether the trial protocol submitted with this manuscript was published before the analysis was undertaken, as I am not able to determine that. The trial protocol was published in February 14, 2018, and the data collection begun in March, 2018. The study protocol was then published before the study analysis was undertake.
