# Supplementary material for: Continuous positive airway pressure to reduce the risk of early peripheral oxygen desaturation after onset of apnoea in children: A double-blind randomised controlled trial
Source: PLoS One. 2021 Oct 1;16(10):e0256950. doi: 10.1371/journal.pone.0256950 (PMC8486132; doi:10.1371/journal.pone.0256950)
Supplement: Attachment — Submitted filename: Response to Reviewers.docx [file pone.0256950.s016.docx]

Initially, we are grateful for all the suggestions. Without a doubt, they praised our paper. All questions were answered directly in the original text and we remain at your disposal for any clarifications that are still needed.

1. Abstract: in the third-from-bottom line, please report the exact p-value instead of p<0.05 unless it is very small e.g. p<0.001. Thank you. Corrected in the manuscript

2. Introduction: given your response to the previous round of reviews, it is not correct to say that you measured SpO2 every 30 seconds as indicated in the last paragraph of the introduction; perhaps it is just better to say that you measured time to recovery of pre-apnoea SpO2 levels. (The way this has been described in the paragraph in the methods just above 'statistical analysis' seems fine though.). Thank you. Corrected in the manuscript.

Methods:

3. similar to the comment above, given your response it is misleading to say in the 'materials and methods' section that you recorded SpO2 values every 30 seconds, if you were continuously checking SpO2 to determine the actual time in seconds to recovery to pre-apnoea levels, unless you were doing this in 30-second increments, which you have indicated in the response that you were not doing. Corrected in the manuscript.

4. indicating the number of children in the first line of materials and methods is actually a non-standard reporting practice, as this is actually a result. It would have been sufficient to say 'we conducted a randomised double blind controlled parallel group trial in children undergoing surgery at…' Thank you. Corrected in the manuscript.

5. the methods also need to describe the treatment received by the control group. You could add this immediately after describing the intervention, just before where you describe the recruitment in the surgical wards. Added in the manuscript.

6. the description of the sample size calculation is still incomplete and not reproducible. Now that it is clearer that this was a time-to-event outcome, for 80% power to detect a 50% relative reduction in hazard you would expect to observe 66 events (this is not the sample size); however your sample size calculation should further indicate what the expected proportions remaining event-free at 300 seconds, given that the sample size would be obtained by dividing the expected number of events by [1 - ((S1+S2)/2)] where S1 and S2 are the proportions in the control and intervention groups respectively that remain event-free at 300 seconds. If under standard care S1 participants remain event free at 300 seconds, then for a relative reduction in hazard of 50%, S2 = S1 x exp(0.5). S1 should have been determined based on information available before this study. Thank you for the support. To recalculate the sample size, a 5% survival rate was admitted in the control group at the end of the study. Thus, S1 = 0.05. Therefore, S2= 0.05^0.5^ = sqrt(0.05) = 0.2236 and the sample size would be approximately equal to 76 patients**:** ([1 - (S1+S2)/2)] = [1 – (0.05 +0.2236)/2] = [1 - 0.2736/2] = 1 - 0.1368 = 0.8632, n = 66/0.8632 = 76.45968). With a sample of 68 patients, the power of the study is 72,4%. During the discussion with our hospital's anaesthesiology clinical staff to limit the observation time to 5 min due to ethical issues, we decided to use data previously obtained in our own unit (pilot study) before patient collection to determine the 5% threshold.

7. it is unclear where the chi-squared tests, Fisher's exact test, students t-test etc referred to in the statistical methods were applied. In any case, they don't appear to be necessary at any stage of the analysis. Thank you. Corrected in the manuscript.

8. what you report in the methods regarding the test for normality is actually a result; instead it would have been sufficient to indicate here that for continuous measures, the test for normality was conducted and you reported means and SDs of normally distributed variables or medians and IQRs for non-normal quantities - without alluding to the results of this test. Thank you. Corrected in the manuscript.

Results

9. the key to figure 3 doesn't enable the reader to distinguish between the intervention and control group. Figure corrected

10. Method section: The authors should specify whether children with genetic syndromes, acute and chronic cardiovascular and neurological diseases were included or excluded from the study. Thank you. Our population was formed by children with physical status I or II according to the American Society of Anesthesiologists classification. The only four children (3 in the CPAP group and 1 control) with ASA physical status equal to II had controlled asthma. Children with genetic syndromes, acute and chronic cardiovascular and neurological diseases were not included.

page 11: please correct the following sentences:

11. “Fig 2 shows the plots of Kaplan-Meier curves for groups CPAP and Control" with "Figure 2 shows the Kaplan-Meier curves for the CPAP and control groups” Corrected in the manuscript.

12. ”between-group with between groups” Corrected in the manuscript.

13. The study is well-designed and controlled, appearing to be scientifically sound. The concerns of the prior reviewers seem to have been adequately addressed. Regarding point #9 on power analysis, as any change to the power calculation would be a posteriori I would recommend still reporting the calculations that were performed a priori. If the authors wish to include an additional a posteriori calculation based on the reviewer recommendations to demonstrate the true power of the study, I would suggest stating exactly what happened: the study population size was based on the a priori calculation, but a posteriori a second analysis was performed based on feedback that the initial assumptions required adjustment, and this analysis revealed the study was powered at X% to detect Y difference, or something to that effect. Thank you. Corrected in the manuscript.

Specific comments:

14. Abstract: as a result of deletions, T1 is no longer defined in the abstract, but T2 is. The T1 definition can be appended to the sentence about the primary outcome. Corrected in the manuscript.

15. P7 third paragraph: by ‘cardioscopy’ do you mean electrocardiogram? Yes, that is correct. Modified in the manuscript.

16. P7 third paragraph: if 60% fraction was oxygen, was the rest nitrogen gas (room air)? This should be stated. Information added in the manuscript.

17. P8 first paragraph: change ‘halt’ to ‘halted’ Corrected in the manuscript.

18. P11/Figure 3: why does the control group return to a non-significant difference at 240 seconds? Is this due to patients in the control group getting bag mask ventilation due to falling saturations (post T1 measurement)? If so this figure is misleading and the more appropriate analysis would be to look at only patients who had not yet reached the T1 endpoint when comparing the groups. Thank you. The Figure 3 refers to the measurements made during the T1 fase, after the apnoea onset till the pulse oximetry equals 95%. Once the patient reached a 95% pulse oximetry, the fase T2 fase started and this data is not represented in Figure 3. Our hypothesis is that the lack of significance after 240 seconds is because the groups tend to match up from that moment on, that is, the performance of CPAP would have the greatest impact between 60 and 210 seconds. After 240 seconds, those patients in the control group who did not desaturate would probably not benefit from using CPAP, showing that the benefit of the intervention would be in those patients more likely to desaturate early.

19. P13 second paragraph: change ‘define the airway’ to ‘secure the airway’ Corrected in the manuscript.

20. P13 last word: change overpasses to surpasses Corrected in the manuscript.

21. P14 second paragraph: you state that other studies ‘[show] improvements in outcomes by the use of positive pressure’. Is there definitive evidence that outcomes are improved with CPAP? I can see reduced rates of transient hypoxia during apnea, as you have shown here, but I don’t believe there is evidence that meaningful case outcomes such as postop oxygen requirements, reintubation requirements, etc. are reduced. Thus I would recommend defining what outcomes, if any, are improved. Thank you. Information added in the manuscript.

22. P14 third paragraph: change ‘being one’ to ‘one being’ Corrected in the manuscript.

23. P14 third paragraph: change ‘showed’ to ‘shown’ Corrected in the manuscript.

24. P14 fourth paragraph: change ‘hold the small airways opened’ to ‘prevent atelectasis’ Altered in the manuscript.

P15 first paragraph: I would change the word ‘threatening’ to something else, maybe ‘the impairment of these factors may predispose patients to harm’ or delete the whole sentence Deleted in the manuscript.
