# Supplementary material for: Continuous positive airway pressure to reduce the risk of early peripheral oxygen desaturation after onset of apnoea in children: A double-blind randomised controlled trial
Source: PLoS One. 2021 Oct 1;16(10):e0256950. doi: 10.1371/journal.pone.0256950 (PMC8486132; doi:10.1371/journal.pone.0256950)
Supplement: Attachment — Submitted filename: Response to Reviewers.docx [file pone.0256950.s017.docx]

We are grateful for all the suggestions. Without a doubt, they praised our paper. We’ve made some minor adjustments in the text: an excerpt that was previously in the methods was moved to the discussion as we understand that this would be more appropriate since it refers to the limitations of the study, and the reference number 18 was updated. All questions were answered directly in the original text and we remain at your disposal for any clarifications that are still needed.

The authors have responded very well to previous comments. There is still one issue that the authors need to revisit. The sample size calculation is still insufficiently described. First, I would recommend that the authors include a reference to the Schoenfeld’s procedure which they used in the original sample size calculation, and also state the parameters/assumptions that went into the calculation. In the 'revised' calculation which is included in the authors' response to previous comments, the formula is incorrectly applied, specifically where the authors divide S2 by 2 (the correct application is to first sum up S1 and S2 and then divide this sum by 2; the result of this calculation is then subtracted from 1; and finally, the result of this second part of the calculation is multiplied by the expected number of events (i.e. 66 expected events for 50% relative reduction at 80% power) to obtain the overall sample size. Thank you for the opportunity. Unfortunately, S2= 0.050.5 was a printing error made by us in the last Response to Reviewers. It was not our intention to and we did not divide S2 by 2. Below we show in detail how the initial and revised sample calculations were performed.

**1. Assumptions for original sample size calculations:**

1.1 We want to able to detect a 50% reduction in the hazard of the experimental group with a power of 80% and a significance level of 0.05.

1.2 We assumed no censoring.

The reference for Schoenfeld procedure used in original sample size calculation comes from the following Stata’s SE 12.1 command that we used to calculate the sample size:

**stpower logrank, hratio(0.5) power(0.8) schoenfeld**

Note: By default, stpower assumes an equal allocation design (input parameter: p1 = 0.5000)

Output (original sample size calculation):

**2. Revised calculation of sample size:**

2.1 We want to able to detect a 50% reduction in the hazard of the experimental group with a power of 80% and a significance level of 0.05.

2.2 We assumed that 5% of the subjects in the control group were expected to survive to end of the study (300 seconds).

Under these assumptions, the required sample size should be:

$$N=\frac{E}{p_{E}}$$

E = estimated number of events (E = 66 from the output above) and $p_{E}$= 1– (S_1_ + S_2_)/2. Now,

S_2_ = S_1_^HR^ = 0.05^0.5^ = sqrt(0.05) = 0.2236. So, $p_{E}$= 0.8632 and the sample size *N* ≅ 76.

This result is confirmed by the following Stata’s stpower output:

**stpower logrank 0.05, hratio(0.5) power(0.8) Schoenfeld**

Output:

The actual study sample was 68 patients. With this sample size we achieved 72,4% power to detect a 50% reduction in the hazard of the experimental group, with a 5% significance level.

The reference for Schoenfeld’s procedure (formula) used in original sample size calculation is found in *An Introduction to Survival Analysis Using Stata*, by Mario Cleves et al. Third Edition, 2010, Stata Press, page 338. Here, the author refers to a Schoenfeld’s paper (The Asymptotic Properties of Nonparametric Tests for Comparing Survival Distributions *Biometrika* 68:316-319) as the source of the formula used in the sample calculations.

This procedure is an option in the following Stata’s SE 12.1 command that we used to calculate the sample size.

Also, please refer to the sample size calculation as such, and not 'study population size’. Altered in the manuscript

In response to previous reviews, the authors removed mentions of chi-squared tests, Fisher's exact test, students t-test etc. Mentions of Shapiro–Wilk test should also be moved. Altered in the manuscript

A reference to the Benjamini–Hochberg procedure should also be included, along with a brief explanation of how it adjusts the p-value to avoid type 1 error. A reference to the Benjamini–Hochberg procedure could be their original article: Benjamini, Y., and Hochberg, Y. (1995). Controlling the false discovery rate: a practical and powerful approach to multiple testing. *Journal of the Royal Statistical Society Series B*, 57, 289–300. Briefly, this method is one of several for dealing with the so-called multiplicity of hypothesis tests problem (http://www.biostathandbook.com/multiplecomparisons.html). When there are several hypotheses to be tested with the data from a particular study, the problem of the occurrence of false positives (type I error) arises. The criticism of many more traditional methods is that they greatly diminish the chance of rejecting a null hypothesis. This also leads to an unwanted increase in false negatives. Benjamini-Hochberg's proposal is not to try to reduce the chance of a false negative so drastically, but to accept a certain proportion of false positives. From there, it defines a procedure to define which null hypotheses among the rejected ones should really be rejected (true positives). An explanation of how it adjusts the p-value to avoid type 1 error can be find at [https://www.statisticshowto.datasciencecentral.com/benjamini-hochberg-procedure/](https://www.researchgate.net/deref/https%3A%2F%2Fwww.statisticshowto.datasciencecentral.com%2Fbenjamini-hochberg-procedure%2F).
